# Supplementary figures and images for: Turnover of retroelements and satellite DNA drives centromere reorganization over short evolutionary timescales in Drosophila
Source: PLoS Biol. 2024 Nov 21;22(11):e3002911. doi: 10.1371/journal.pbio.3002911 (PMC11620609; doi:10.1371/journal.pbio.3002911)

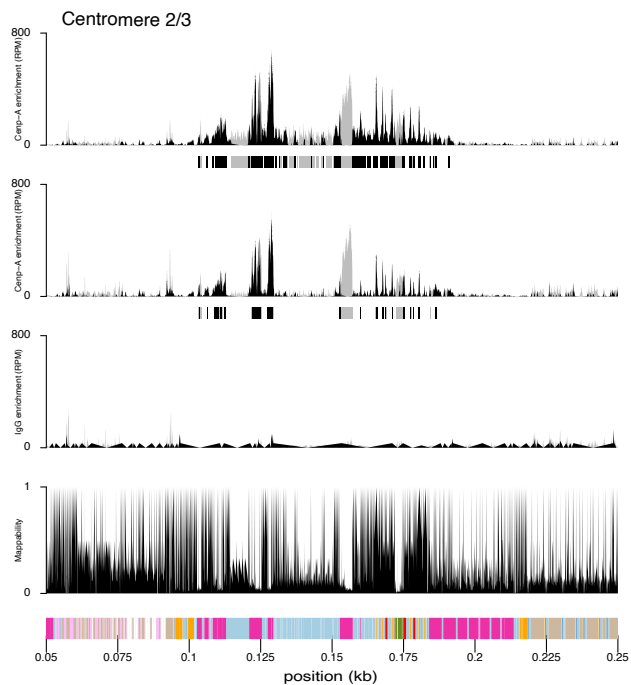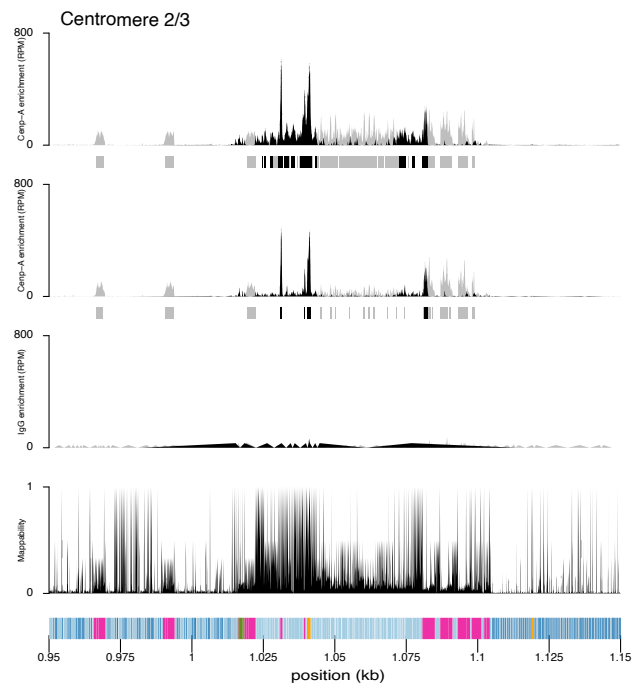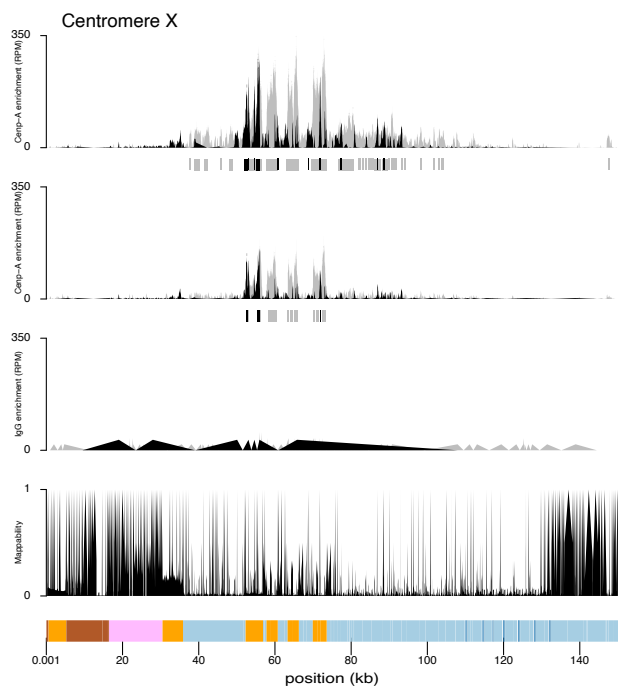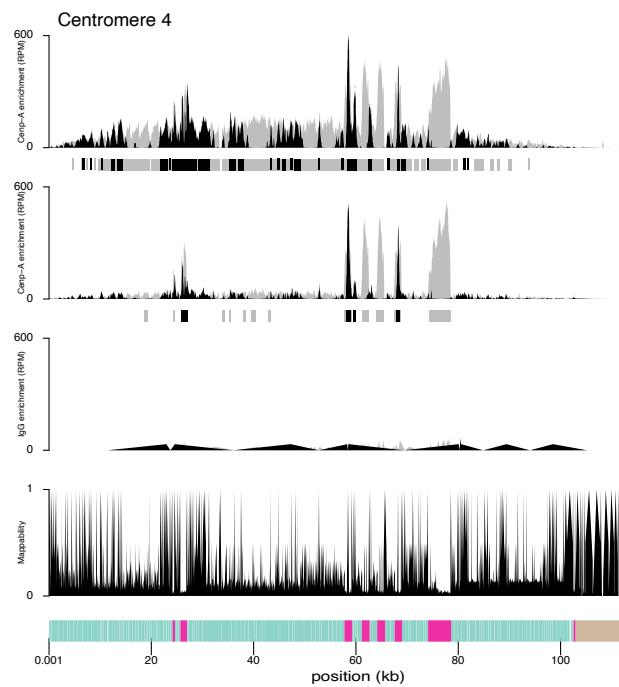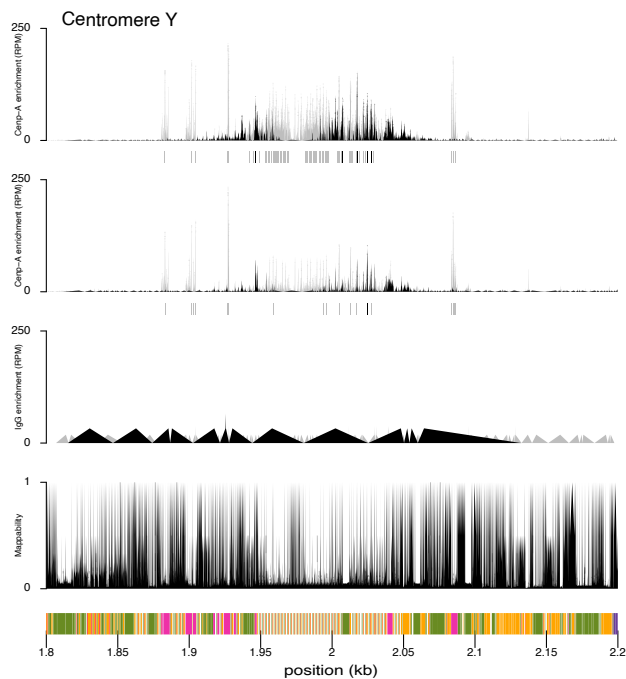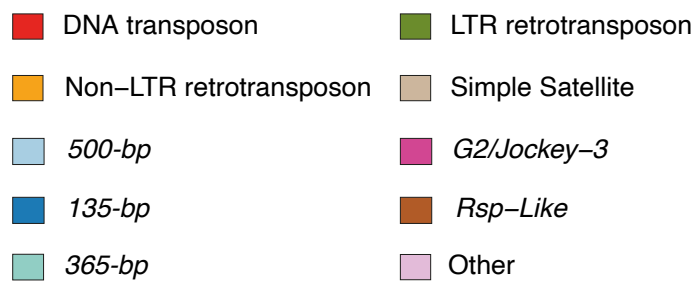

Supplement: S1 Fig — simulans. The y-axis represents the normalized CENP-A or IgG enrichment in RPM. Black and gray plotted lines represent the enrichment based on uniquely mapping and all reads (including multi-mappers), respectively. The black and gray tracks below each plot correspond to MACS2 peaks showing significantly enriched regions based on the uniquely mapping and all reads (including multi-mappers), respectively. The precise locations of all peaks are listed in S1 Table. The colored cytoband at the bottom of the plot shows the repeat organization. The color code is shown in the legend at the bottom of the figure. The data underlying this figure can be found at https://doi.org/10.5061/dryad.1zcrjdg2g [40]. (PDF) [file pbio.3002911.s001.pdf]

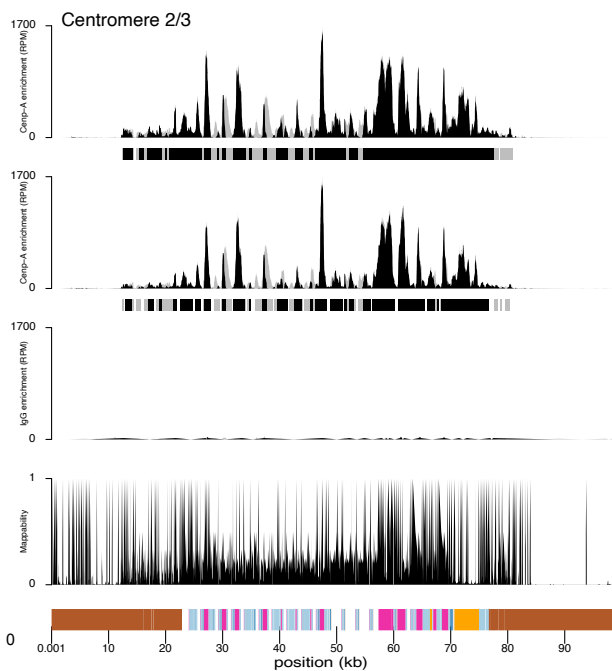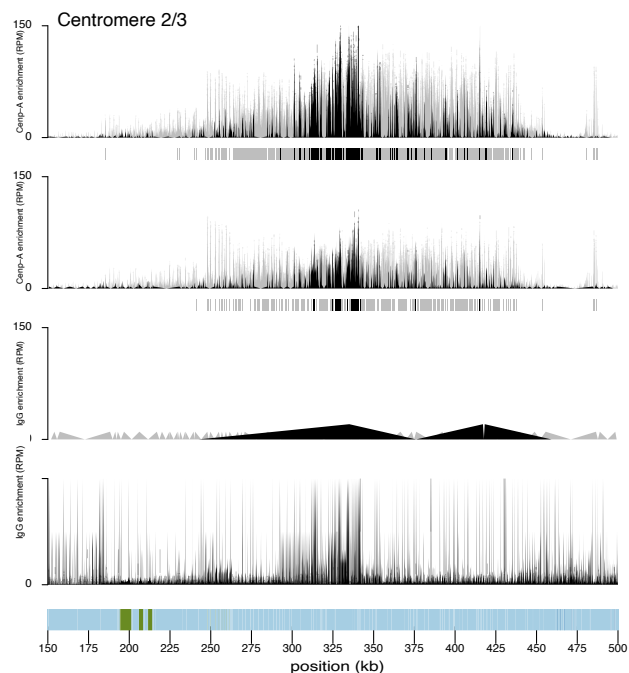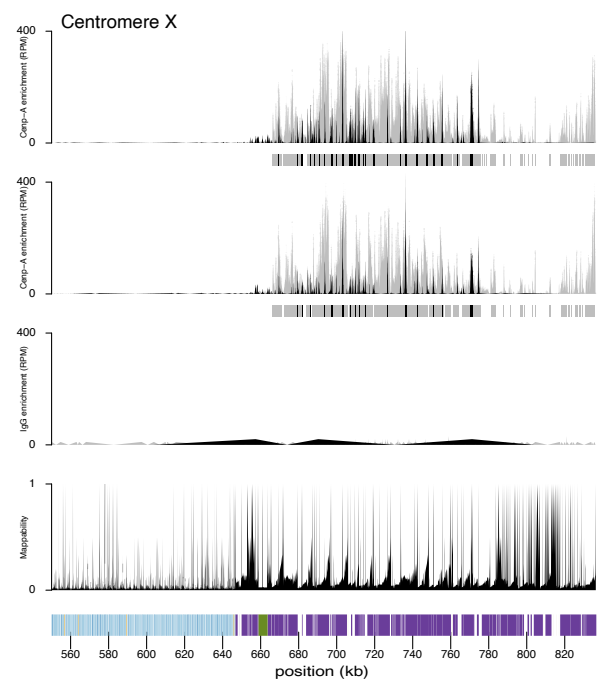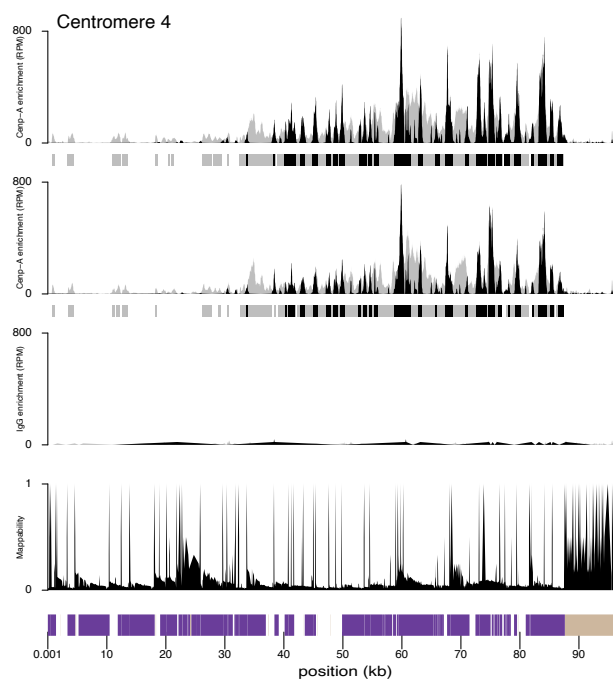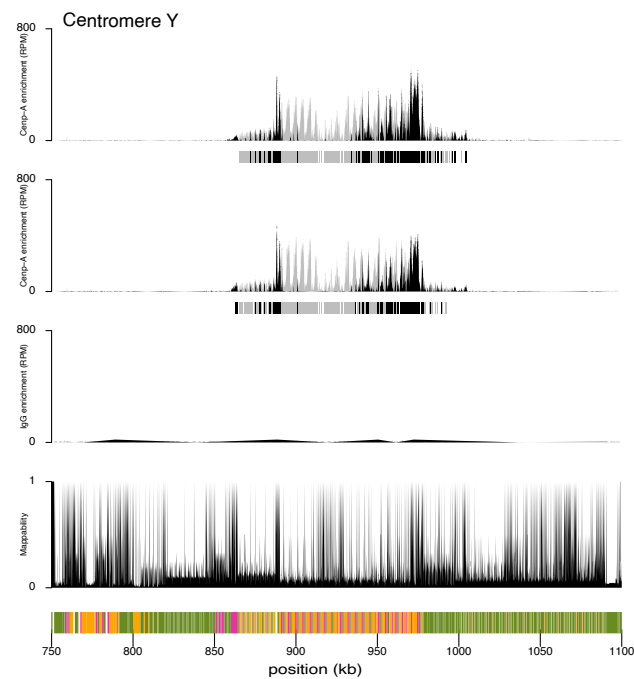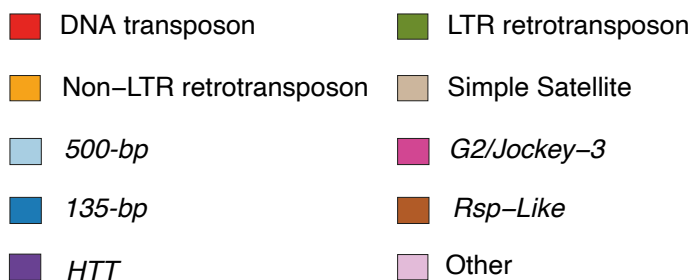

Supplement: S2 Fig — sechellia. The y-axis represents the normalized CENP-A or IgG enrichment in RPM. Black and gray plotted lines represent the enrichment based on uniquely mapping and all reads (including multi-mappers), respectively. The black and gray tracks below each plot correspond to MACS2 peaks showing significantly enriched regions based on the uniquely mapping and all reads (including multi-mappers), respectively. The precise locations of all peaks are listed in S1 Table. The colored cytoband at the bottom of the plot shows the repeat organization. Color code is shown in the legend at the bottom of the figure. The data underlying this figure can be found at https://doi.org/10.5061/dryad.1zcrjdg2g [40]. (PDF) [file pbio.3002911.s002.pdf]

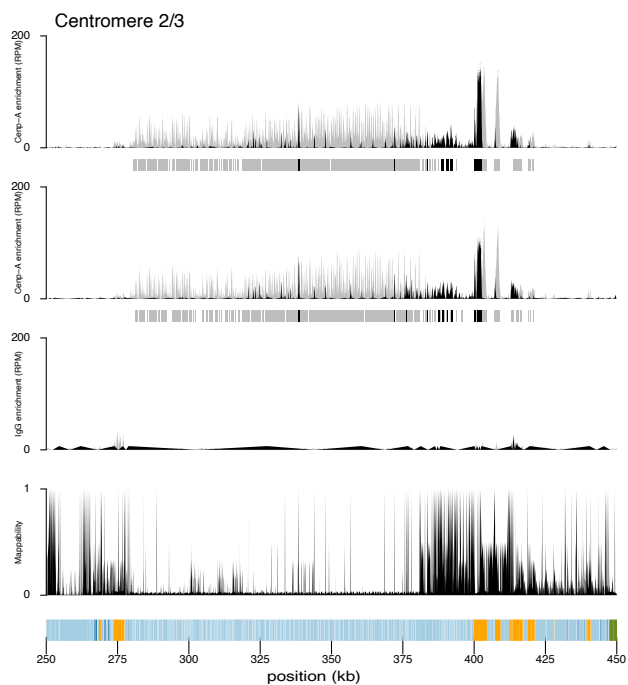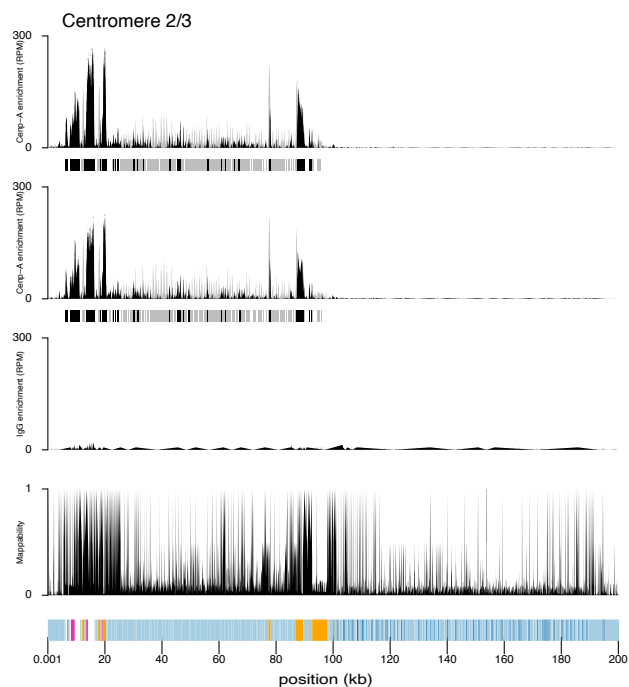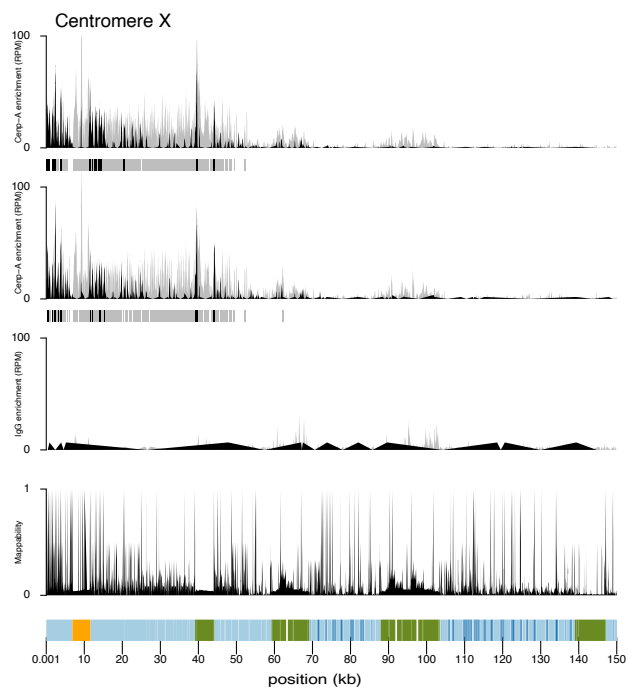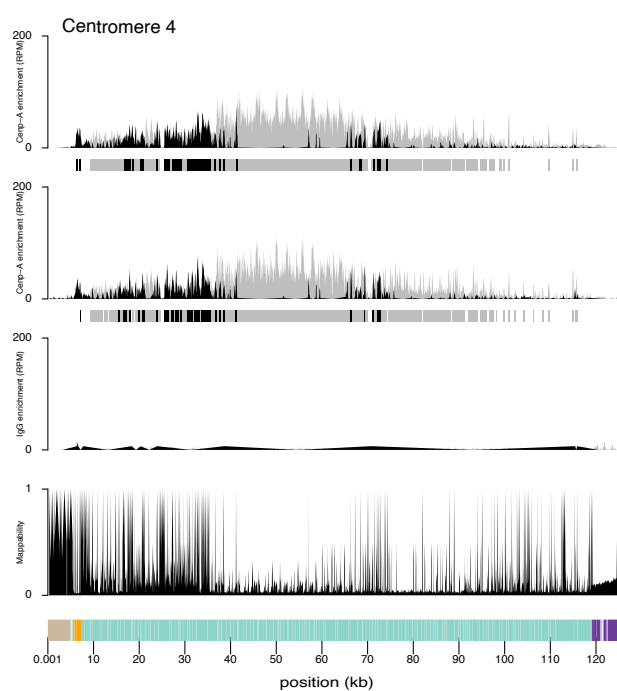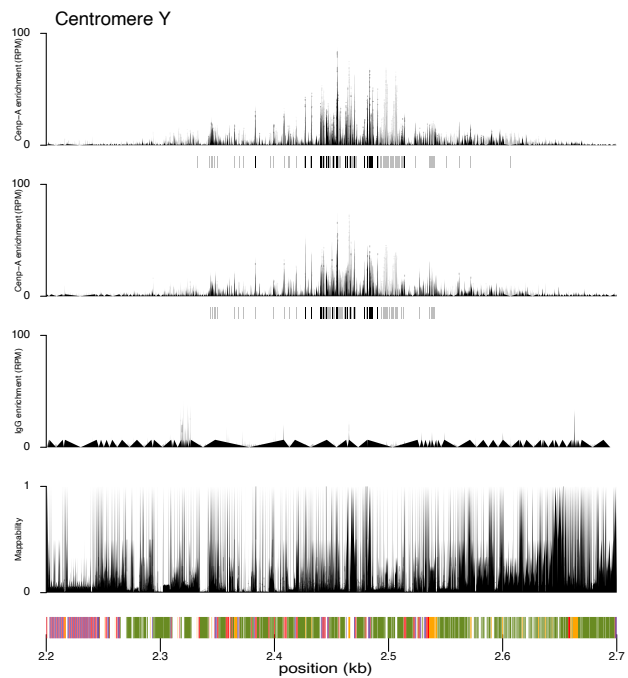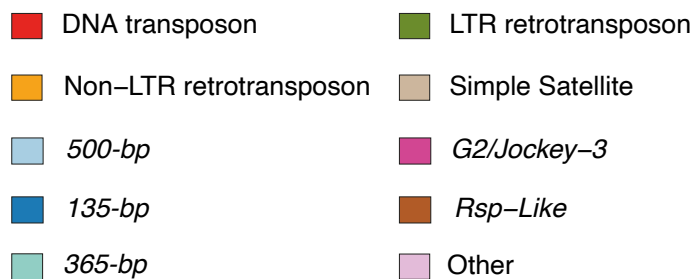

Supplement: S3 Fig — mauritiana. The y-axis represents the normalized CENP-A or IgG enrichment in RPM. Black and gray plotted lines represent the enrichment based on uniquely mapping and all reads (including multi-mappers), respectively. The black and gray tracks below each plot correspond to MACS2 peaks showing significantly enriched regions based on the uniquely mapping and all reads (including multi-mappers), respectively. The precise locations of all peaks are listed in S1 Table. The colored cytoband at the bottom of the plot shows the repeat organization. The color code is shown in the legend at the bottom of the figure. The data underlying this figure can be found at https://doi.org/10.5061/dryad.1zcrjdg2g [40]. (PDF) [file pbio.3002911.s003.pdf]

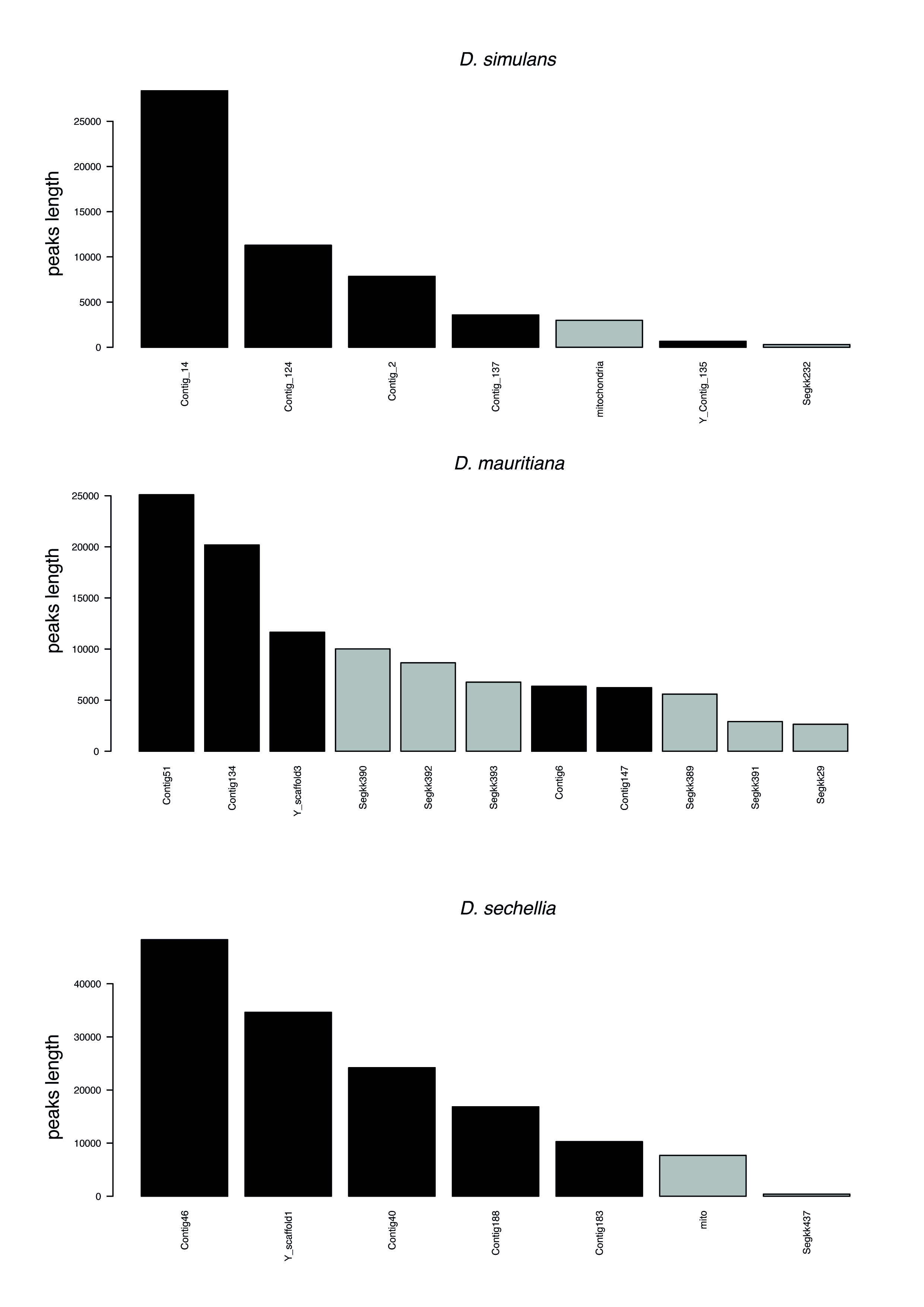

Supplement: S4 Fig — (TIF) [file pbio.3002911.s004.tif]

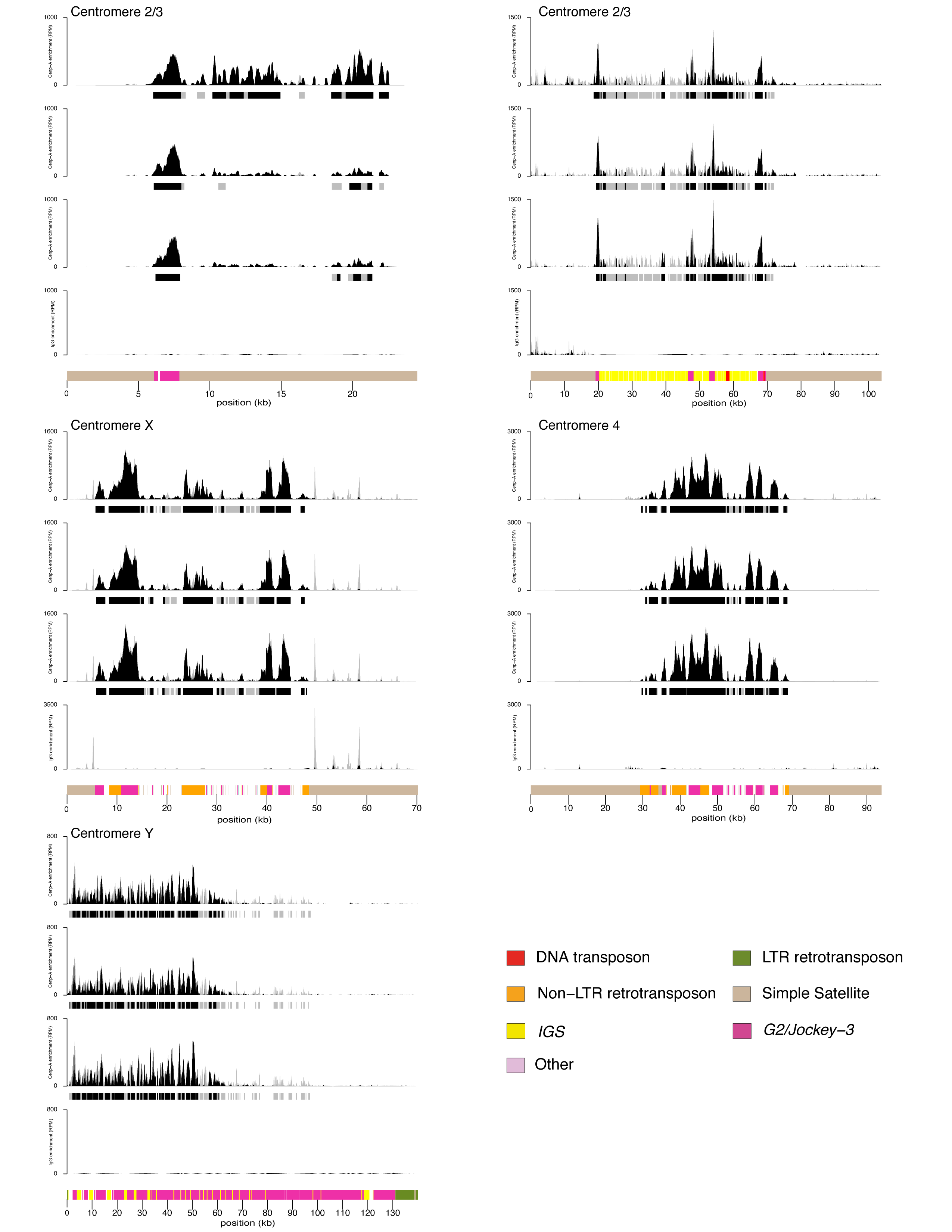

Supplement: S5 Fig — The black and gray tracks below each plot correspond to MACS2 peaks showing significantly enriched regions based on the uniquely mapping and all reads (including multi-mappers), respectively. The precise locations of all peaks are listed in S1 Table. The colored cytoband at the bottom of the plot shows the repeat organization. The color code is shown in the legend at the bottom of the figure. The data underlying this figure can be found at https://doi.org/10.5061/dryad.1zcrjdg2g [40]. (TIF) [file pbio.3002911.s005.tif]

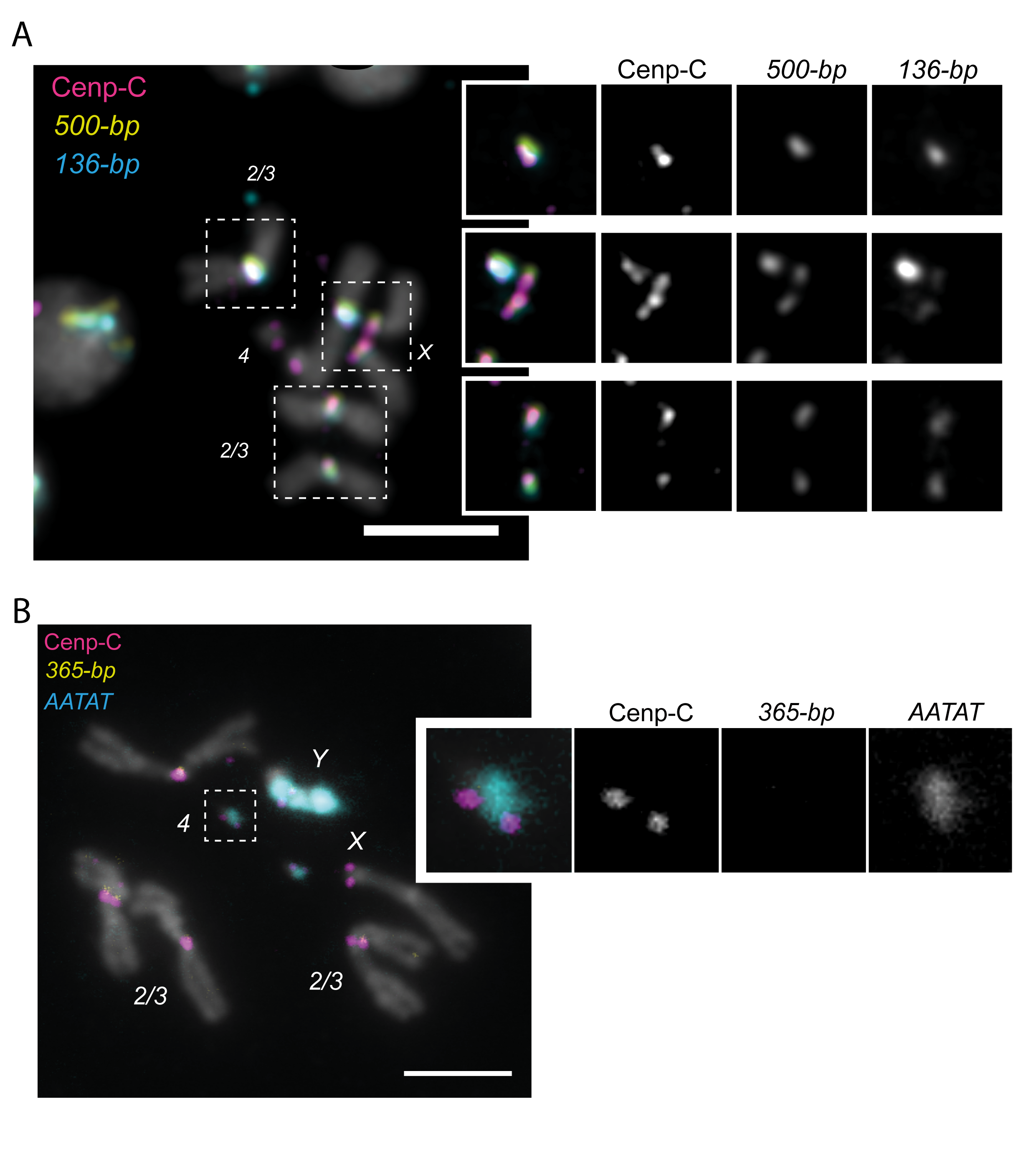

Supplement: S6 Fig — (A) IF-FISH on mitotic chromosomes from the larval brain with CENP-C antibody and 500bp and 136-bp probes. The inset represents a zoom on each centromere. (B) IF-FISH on mitotic chromosomes from the larval brain from D. sechellia with CENP-C antibody and 365-bp and AATAT probes. The inset represents a zoom on the dot chromosome centromere. (TIF) [file pbio.3002911.s006.tif]

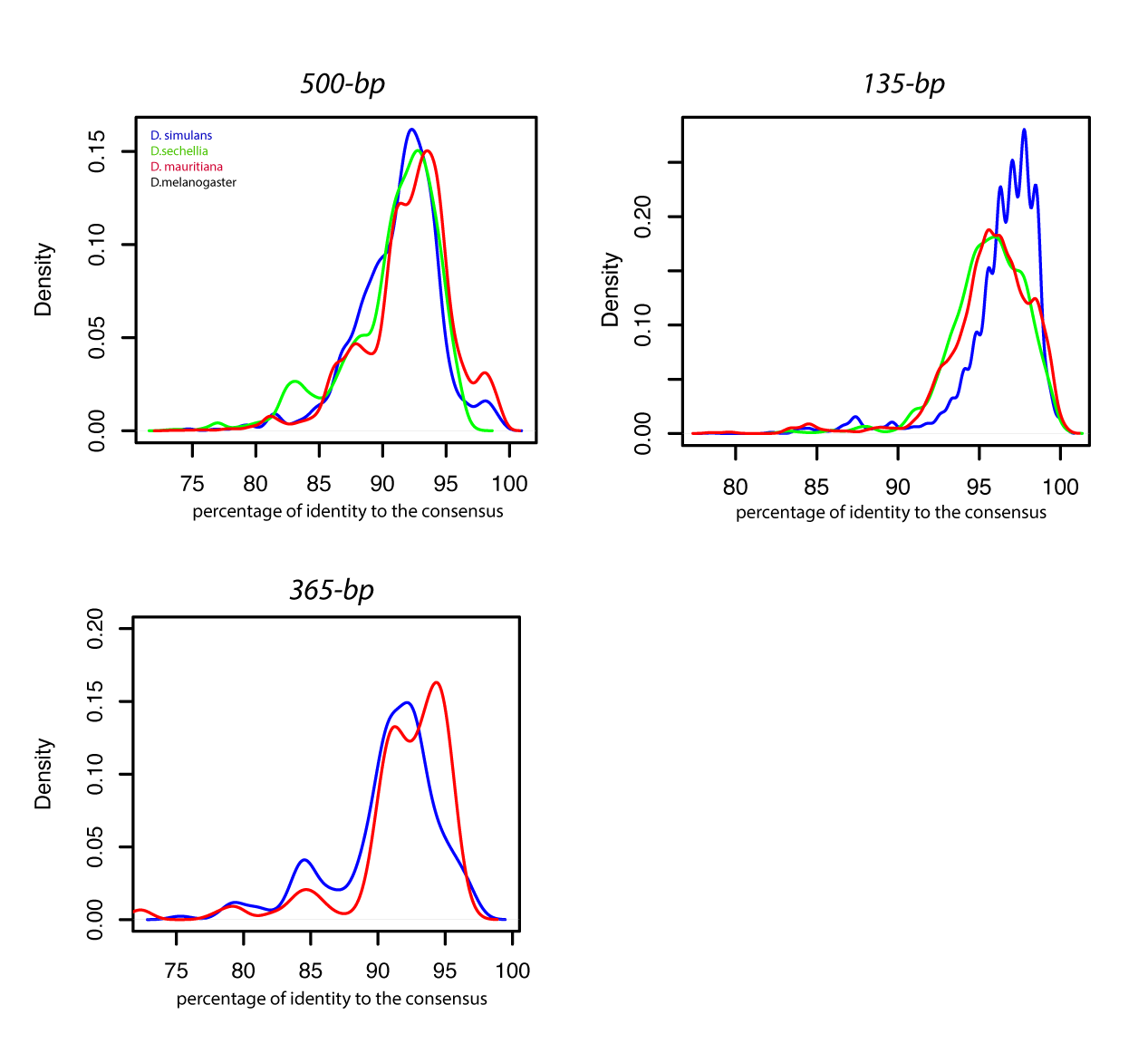

Supplement: S7 Fig — (TIF) [file pbio.3002911.s007.tif]

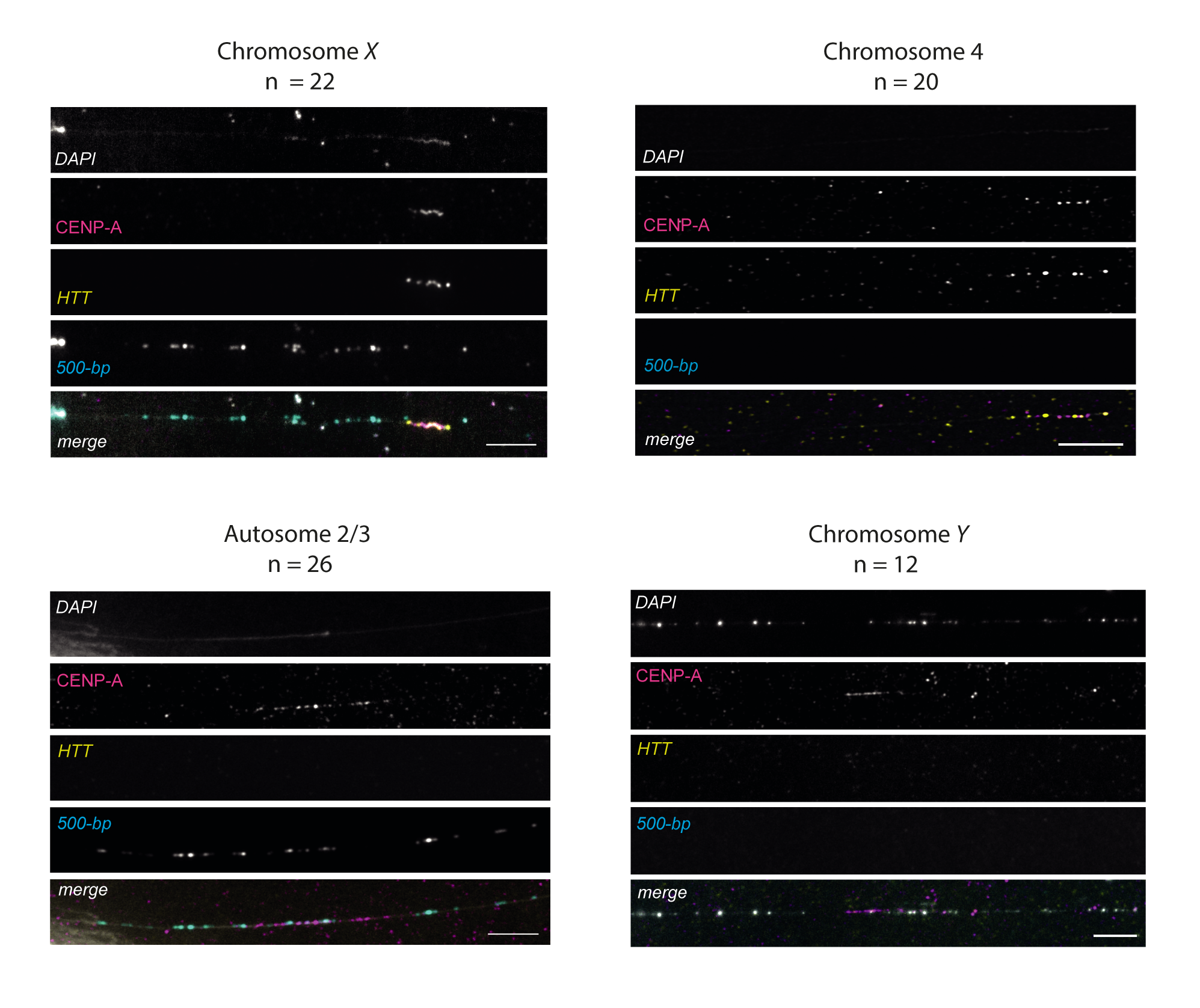

Supplement: S8 Fig — sechellia larval brains with CENP-A antibody and 500-bp and HTT probes. A representative image of each centromere pattern is presented along with the total number of images collected for each pattern. CENP-A is present on the HTT region with or without 500-bp flanking, corresponding to the X and dot chromosome, respectively. CENP-A is also present on a 500-bp region, corresponding to the autosomal centromeres and without 500-bp nearby, consistent with the Y chromosome. (TIF) [file pbio.3002911.s008.tif]

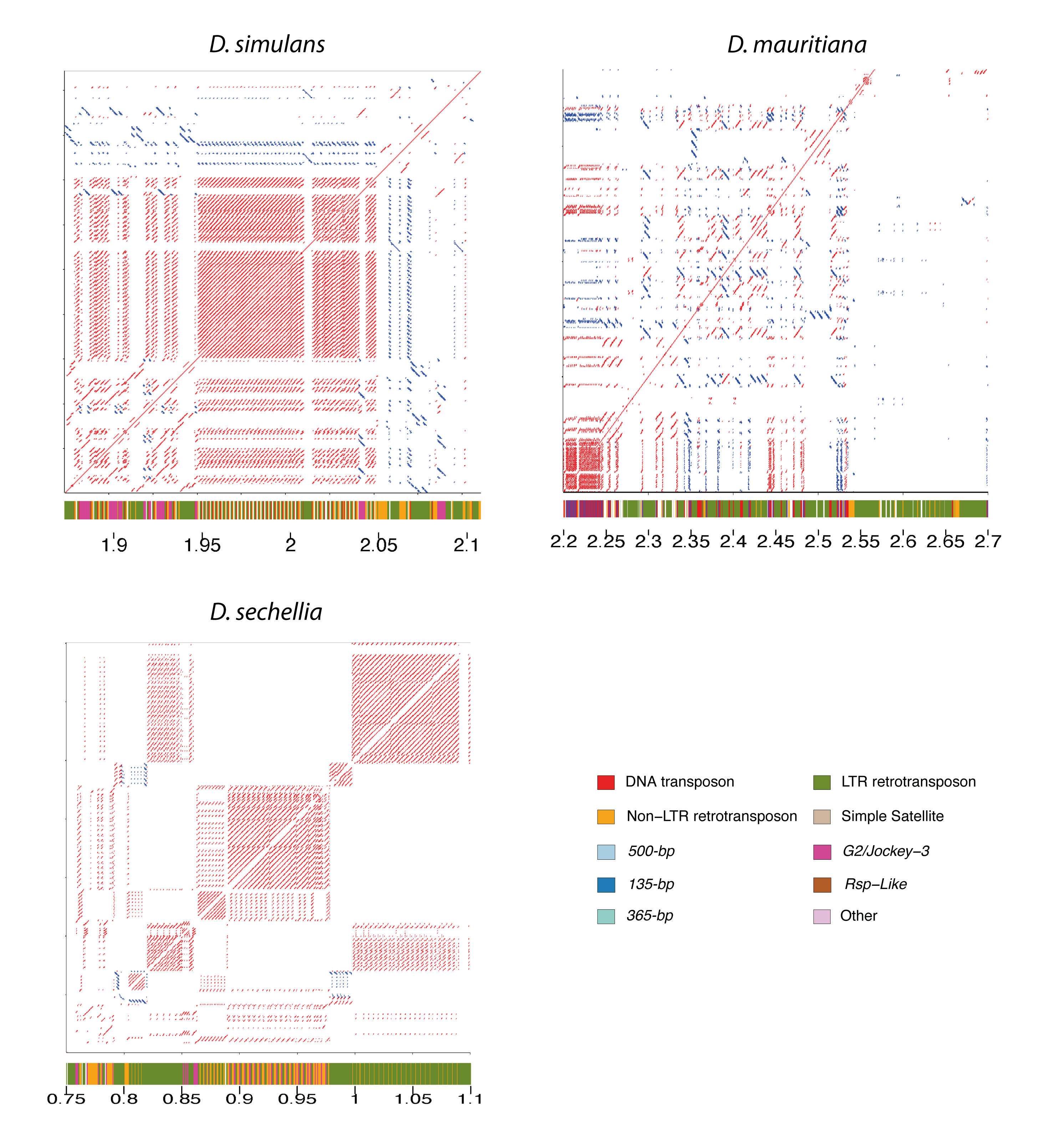

Supplement: S9 Fig — The Dotplot was produced using re-DOT-able with a sliding window of 100 bp. The cytoband below each dotplot represent the repeat composition of the region. The color code is indicated in the legend. (TIF) [file pbio.3002911.s009.tif]

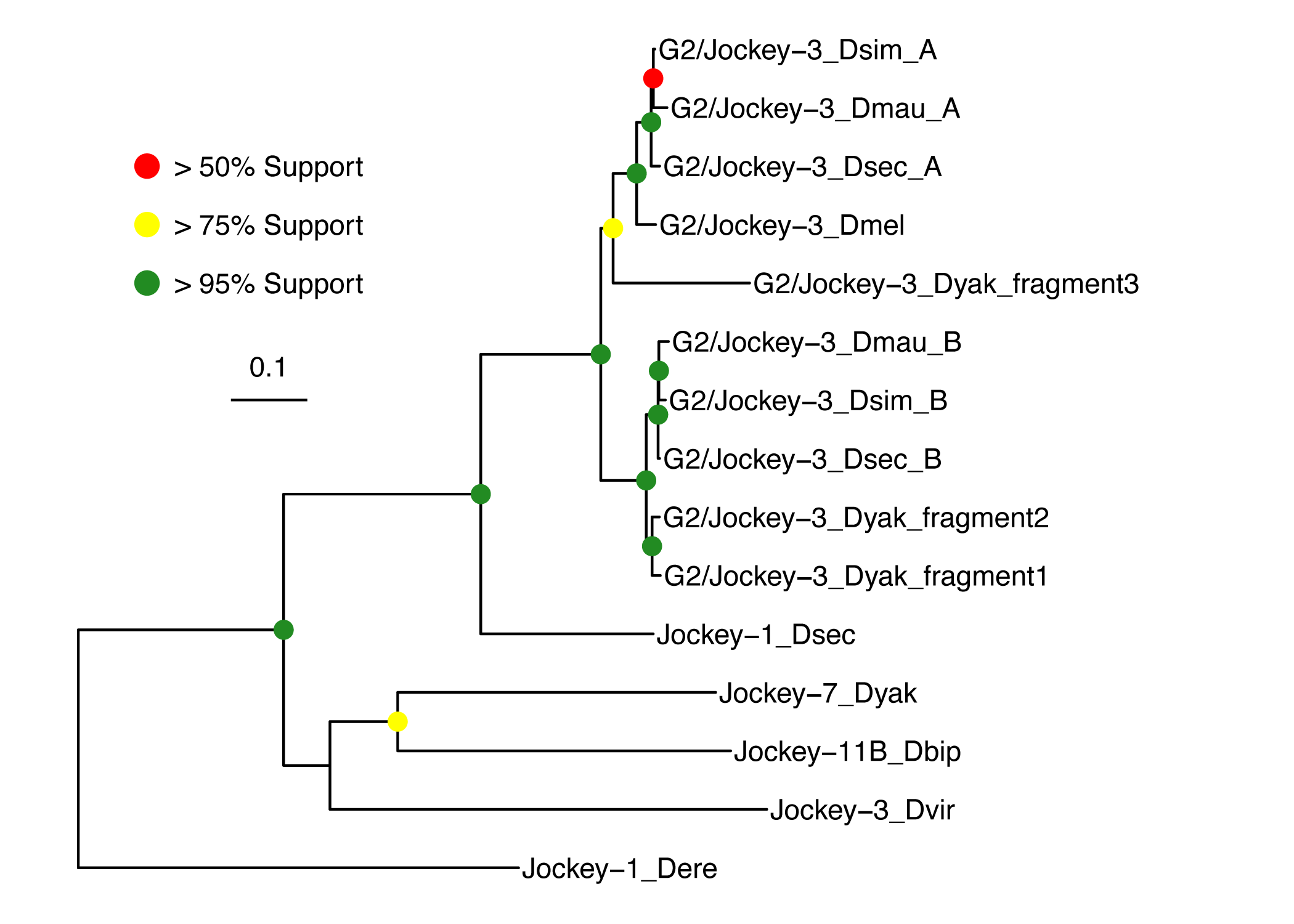

Supplement: S10 Fig — Three D. yakuba fragments which span the >50% of the ORF are also included. The data underlying this figure can be found at https://doi.org/10.5061/dryad.1zcrjdg2g [40]. (TIF) [file pbio.3002911.s010.tif]

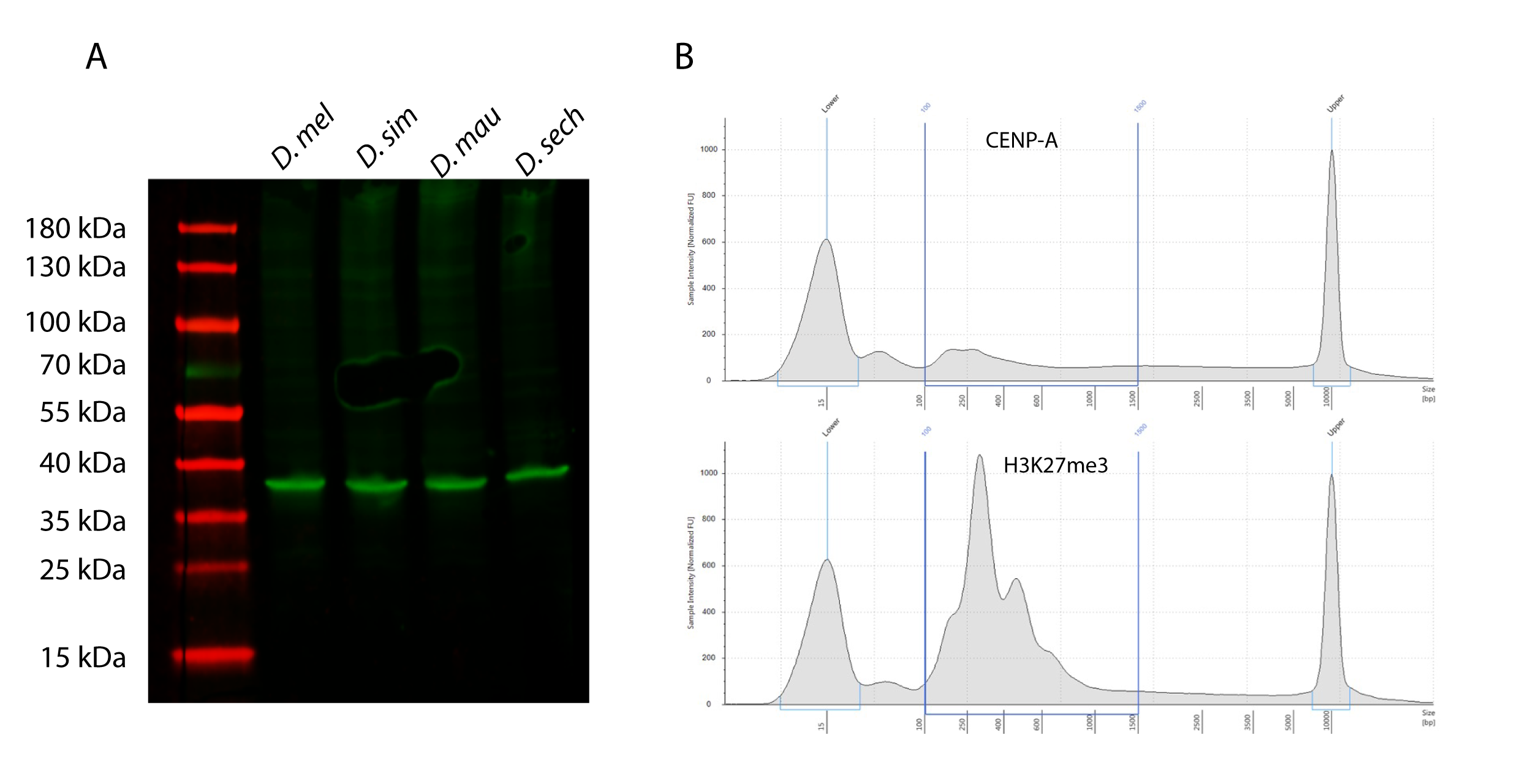

Supplement: S11 Fig — (A) Western blots using our custom-generated CENP-A antibody on samples from all 4 species D. melanogaster clade species. (B) Bioanalyzer profile of the CUT&Tag libraries obtained for our custom-generated CENP-A and H2K27me3 antibodies. (TIF) [file pbio.3002911.s011.tif]
