# Supplementary material for: Turnover of retroelements and satellite DNA drives centromere reorganization over short evolutionary timescales in Drosophila
Source: PLoS Biol. 2024 Nov 21;22(11):e3002911. doi: 10.1371/journal.pbio.3002911 (PMC11620609; doi:10.1371/journal.pbio.3002911)
Supplement: Attachment — Submitted filename: Courret_Response.to.review.2.2024.pdf [file pbio.3002911.s034.pdf]

Thank you for handling our manuscript and for the constructive feedback. We respond to each comment point-by-point below. Our responses are in blue text.

You'll see that reviewer #1 continues to be positive, but still has two requests. One is to do a formal analysis of between-replicate similarity. The other is to incorporate “mappability” into the Figs, either by normalising by a mappability index, or by including a mappability track for visual comparison. Reviewer #2 thinks that the paper is much improved, but still says that you can’t support the “reveals signatures of genetic conflict” aspect of the Title; instead s/he thinks that any mechanistic claims should be moved to the Discussion and clearly flagged as speculative. S/he also has two more minor requests; one is to better contextualise your CUT&Tag results in the Figs, and the other is to drop the inconsistent fiber-IF/FISH from Fig 4D.

Based on the reviews, we are likely to accept this manuscript for publication, provided you satisfactorily address the remaining points raised by the reviewers. Please also make sure to address the following data and other policy-related requests.

We are happy to receive this feedback and hope that you agree that we resolved all remaining concerns.

IMPORTANT - please attend to the following:

a) After discussion with the Academic Editor, we agree with reviewer #2's suggestion that you remove the "conflict" claim from the Title. To make the Title more explicit and appealing to our broader readership, we suggest that you change it to something like "Turnover of retroelements and satellite DNAs results in profound centromere reorganization over short evolutionary timescales in Drosophila species"

Following Reviewer #2 and the editor's suggestion we changed the title to: “**Turnover of retroelements and satellite DNA drives centromere reorganization over short evolutionary timescales in Drosophila**”

b) Please address the remaining requests from the reviewers. In addition, the Academic Editor made the following request about your Discussion: "For the discussion, I suggest that the authors begin with the two major hypotheses to explain rapid evolution 1) lack of purifying selection or 2) positive selection (including that which arises from genetic conflict). If the discussion is framed around these alternatives it will give the authors the opportunity to more clearly lay out what aspects of their data they think relate to genetic conflict as well as other aspects that may reflect lack of purifying selection. For example, the total lack of conservation of centromere sequences must in part reflect an absence of purifying selection on the sequence itself. I think this frame work will also reduce any appearance of bias on the part of the authors towards a conflict hypothesis, while still allowing plenty of space for thoughtful reflection on their results."

Thank you for the suggestion. We agree that this is a better approach. We rearranged the discussion to briefly summarize our findings in the context of centromere evolution in the field and then layout two hypotheses for the rapid turnover: 1) relaxed constraints on centromeric DNA; and 2) selection of the classical variety where certain DNA sequences are favored or from genetic conflicts where certain DNA sequences are selfish. We then discuss our data with respect to these hypotheses. We make it clear that our speculation is that conflicts might drive centromere turnover and that distinguishing between these hypotheses will require experimental and evolutionary genetic evidence.

In addition to restructuring the Discussion and changing the title, we also added a qualifying sentence to the abstract, and changed the Figure 7 label to 'centromere repeat turnover' instead of 'centromere repeat conflicts'.

c) Please address my Data Policy requests below; specifically, we need you to supply the numerical values underlying Figs 1ABDEGH, 2AB, 3AB, 4AB, 5ABC, 6A (treefile), S1, S2, S3, S4, S5, S7, S9, S10 (treefile), S11B, either as a supplementary data file or as a permanent DOI'd deposition. I note that you have plans for a Dryad deposition, but we will need to check this. Please could you complete this deposition with the data and code needed to recreate the Figure?

We deposited all of the data file needed to recreate the figures in a Dryad repository (https://doi.org/10.5061/dryad.1zcrjdg2g). The submission is set to be publicly released after Dryad curators process our submission. Before the link becomes active, the submission can be viewed here: http://datadryad.org/stash/share/386hTahwwz1ph3SDDMxEc8nvNk-FlGBW22LjuUph9zM.

d) Please cite the location of the data clearly in all relevant main and supplementary Figure legends, e.g. "The data underlying this Figure can be found in S1 Data" or "The data underlying this Figure can be found in
https://urldefense.com/v3/__https://zenodo.org/records/XXXXXXXX__;!!CGUSO5OYRnA7CQ!aFRSuFwpiJMpnUyY3v7Qs48QGdSRZfHg-fzYutg4pVlkTZsxf-zgxNN2RpC-bxyZ8IKXi9FB4WYUU0bmHtZDBw$

We now indicate the location of the data in the legend of each main and supplementary Figures.

e) Please make any custom code available, either as a supplementary file or as part of your data deposition. You already mention a Github deposition, but this is not currently accessible (we will need to check it). Also, because Github depositions can be readily changed or deleted, please make a permanent DOI'd copy (e.g. in Zenodo, or as part of your planned Dryad deposition) and provide this URL (see below).

In addition to the public Github repository with our code, we also make a permanent copy of the code available in our Dryad repository (https://doi.org/10.5061/dryad.1zcrjdg2g)

REVIEWERS' COMMENTS:

Reviewer #1:

The revised manuscript by Courret et al. addresses many of my prior concerns, and I thank the authors for their thoughtful and detailed responses to my earlier critiques. However, I still have two lingering comments about the CENP-A CUT&Tag analysis:

First, while the authors generate replicate CENP-A CUT&Tag libraries for each species, there is no formal analysis of similarity across replicate samples. Visual similarity across replicates is apparent, but this could simply reflect variation in mappabilty across the centromeric locus (see my second major point below). I think the paper would benefit from a more formal analysis showing similarity across replicate libraries. For instance, a PC analysis of CUT&Tag sequences could help demonstrate that CENP-A CUT&Tag findings are reproducible and robust to technical artifacts.

Thank you for your feedback. We did conduct formal analyses of replicability across replicate CUT&Tag datasets. We used an IDR to determine the statistically significant overlapping peaks between replicates and plot only those peaks that are replicable are in our analysis. This analysis is described in the methods under 'Centromere identification', in Figure S4, and Table S1.

Second, the CENP-A association landscape plots in Figs 1-5 are difficult to interpret. The rationale for independently surveying both uniquely mapping reads and all reads mapping to these regions is logical and sound, but the resulting CENP-A association and peak profiles are likely largely dictated by the "mappability" of these genomic regions (i.e., the density of sequences within the reference where reads can be uniquely assigned). For example, in regions where CENP-A associates with centromeric chromatin but reads cannot be uniquely mapped, peaks will be inferred in the multimapping, but not the uniquely mapping, data. Conversely, peaks inferred from pileups of uniquely mapping reads may simply represent regions where reads can be placed uniquely, rather than bona fide sites of CENP-A enrichment. In view of these limitations, CENP-A peak calling ought to be standardized by a "mappability" criterion, in addition to the number of sequenced reads. Alternatively, it could be helpful to include a track that highlights centromere regions where reads can be uniquely mapped.

Thank you for the suggestion. We estimated mappability using the software GenMap which is based on a kmer approach and used 150-mers to match our read length. We now add mappability tracks to Figures S1-S3 to allow for a direct comparison with the CENP-A tracks These data show that there are mappable regions within each centromere.

Despite these criticisms, the author's overall robust approach, which relies on the summation of evidence across multiple bioinformatic methods and cytology experiments, provides assurance that major conclusions are not artifacts of biases or limitations of one analysis. For this reason, I am overall enthusiastic about this work. I also think the authors' focused edits to the manuscript help to more strongly emphasize the novel evolutionary trends they uncover in centromere architecture.

Thank you for the positive feedback.

In addition to these points, I have a few minor comments:

Page 3, Lines 64-65 - "The overexpression of CENP-A…": This sentence stands out as a non-sequitur to me and could (optionally) be removed.
We agree, thanks for the suggestion. We removed this sentence.

Page 4, Lines 90-92: This is true for some, but not all, mouse centromeres. See, e.g., Chmatal et al. 2014 Curr Biology (PMID: 25242031).
Thank you for suggesting this clarification. We changed the sentence to read: “For example, some mouse centromeres with larger satellite DNA arrays recruit more centromeric proteins and thus increase their transmission through female meiosis.”

Page 5, Lines 125-127: "These species diverged…" It may be helpful to present divergence times in generations, rather than years, to frame the extent of divergence across the surveyed Drosopholids on a common scale with other taxa. This suggestion also carries over to the comparative discussion presented on lines 493-498. Surely 2.4MY of Drosophila divergence equates to far more generations than represented across 7 MY of great ape divergence, possibly accounting for the relative conservation of centromere satellite sequence architecture in the latter!

Thank you for this insightful point. Indeed, Drosophilids have many more generations per year compared to great apes. We've now added the generation count for *Drosophila* in lines 125-127. However, since estimating the number of generations for other species can be more challenging and direct comparisons between species are complicated, we decided to remove the divergence times in lines 493-498 to prevent any potential confusion.

Page 12, line 348 - Typo: space between "100" and "%" can be deleted.
Done, thanks.

Line 516 - Typo: Mus musculus
Done, thanks.

Lines 613-614 - "Lastly, the high duplication rates of centromere…" Here, the word centromere should be plural. I also find the statement that centromeres have "high duplication rates" a bit

exclusionary, as centromeres also have high deletion and single nucleotide mutation rates. Perhaps it would be more precise to modify this sentence to "Lastly, the high mutation rates of centromeres…"
Thanks for making this point. We agree and made the change: “Lastly, the high mutation rates at centromeres might further promote the birth and turnover of centromeric satellites”

Figure 1. It is not apparent what the two plots in panels A, D, and G represent. In all cases, both plots are labeled "Autosome 2/3" and show distinct repeat composition and peak positions.

Thanks for pointing out that this is unclear. In short, we do not have a T2T assembly and the similar composition of chromosomes 2 and 3 centromeres mean that we cannot distinguish between them at the assembly or cytological level, so we designate the label Autosome 2/3. We added: “The label 'Autosome 2/3' indicates that we cannot distinguish between the 2nd and 3rd chromosome centromeres.” to the figure legend to clarify.

Figure 7. The blue color assigned to the 500bp satellite sequence in the figure legend does not correspond to the blue in the diagram.

Thank you for alerting us to this! We corrected the color in the legend.

Reviewer #2:

The revised manuscript by Larracuente and colleagues addressed many of the presentation and text related issues raised in the first round of revision. It is much clearer now what experiments were done. The way experiments are presented in the main text is also much improved, and the result section generally matches what the experiments test and the data show. (See several remaining issues below.) In its current form, I believe the datasets produced by the authors will be of high interest to researchers interested in centromere and chromosome evolution.

However, the data does not allow the authors to make many of the mechanistic claims in the paper, and indeed these claims are not directly tested against clear null hypotheses. For instance, the current title of the manuscript is not supported by experiments. A more precise title would be "Rapid turnover of centromeric DNA in Drosophila". Throughout, claims regarding any oogenesis-specific effect (e.g., genetic conflict) should be moved to the Discussion and presented in a way that clarifies their highly speculative nature. Similarly, I am not convinced that the work sheds significant light on how changes to DNA sequence versus protein sequence regulate centromere function & evolution, or as to the potentially different centromeric functions of TEs versus satellites.

Based on Reviewer #2 and the editor suggestions we changed the title to remove the term “conflict”. The new title is “Turnover of retroelements and satellite DNA drives centromere reorganization over short evolutionary timescales in Drosophila” .

In addition, we rearranged the discussion to briefly summarize our findings in the context of centromere evolution in the field and then layout two hypotheses: 1) relaxed constraints on centromeric DNA; and 2) selection of the classical variety where certain DNA sequences are favored or from genetic conflicts where certain DNA sequences are selfish. We then discuss our data with respect to these hypotheses. We speculate that conflicts might drive centromere turnover and that distinguishing between these hypotheses will require experimental and evolutionary genetic evidence.

To reiterate, I believe the dataset produced by the authors represent a significant addition to what is known about Drosophila centromeres. My concern is with regards to the mechanistic claims on centromere evolution. Whether a manuscript stripped off these mechanistic speculation merits publication in PLoS Biology is a question I leave for the editors.

Other points:
- The plots representing the Cenp-A CUT&Tag results (e.g., Fig. 1A,D,G) are still hard to interpret without information that places them in the context of the complete chromosomes. The x-axis in these plots refers to 'position' but lack any context. That would be confusing at best, and probably uninterpretable, especially in a wide-readership journal. This is particularly confusing given that the text also refers to other size ranges (e.g., the paragraph in lines 181-190). The data presentation should make it clear how do the 100-500kb regions that are shown relate to the 60Mb regions described in the text.

Unfortunately, there are no T2T (telomere-to-telomere) assemblies available for Drosophila species. The centromeres are part of contigs disconnected from the chromosome arms, making it impossible to determine their exact positions along the x-axis relative to the entire chromosome.Figure 7A is meant to help readers orient themselves on the chromosome. This schematic reflects the cytological analyses we conducted and is consistently presented alongside the CENP-A CUT&Tag plots with the goal of contextualizing our results and providing clearer spatial understanding.

- The explanation given for the inconsistent data in Fig. 4D (the fiber-IF/FISH) is unsatisfying. If this isn't a representative image, then why is it shown? And if this is the result of a "technical artifact" (lines 338-343), then why are the authors choosing to trust other aspects of the data? If this piece of data is crucial to the authors' story, this needs to be clarified, and analysis of the robustness of this method should be presented. If it is no longer a key part of the story, given that the authors modified their claim of 'truly telocentric' centromeres, then this piece of data should be removed from the current manuscript. Once verified, it could be published together with the results of the ongoing experiments that directly address this hypothesis.

This figure validates the overlap between CENP-A and the telomeric HTT elements, consistent with the immunoFISH results on mitotic chromosomes and our centromeric chromatin profiling. This pattern is reproducible and unambiguous (see quantification in SupTable6). Whether or not there is a very small segment of non-HTT DNA at the chromosome tip does not affect our conclusions. Our data strongly support the assertion that these chromosomes are telocentric. Therefore, we chose to retain these figures and remain transparent about variation in signal continuity at the very ends of the fibers. Such variability is not unusual for this type of data.
