# Supplementary material for: Turnover of retroelements and satellite DNA drives centromere reorganization over short evolutionary timescales in Drosophila
Source: PLoS Biol. 2024 Nov 21;22(11):e3002911. doi: 10.1371/journal.pbio.3002911 (PMC11620609; doi:10.1371/journal.pbio.3002911)
Supplement: Attachment — Submitted filename: Response_to_Reviewers.docx [file pbio.3002911.s032.docx]

We thank the editor and reviewers for their constructive feedback on our manuscript. Please find a revised version of the paper that we submit now for your consideration. A point-by-point response to each reviewer's comment is provided below in blue text) with the key changes to the manuscript summarized (figures and italicized text).

Reviewer #1:

Courret et al. apply a combination of CENP-A chromatin profiling and cytogenetic experiments to assess the sequence-level evolutionary dynamics and TE composition of centromeres in multiple Drosophila species. Their work uncovers new understanding of centromere organization in Drosophila, including the conserved presence of G2-Jockey3 TEs in centromeres of both the D. melanogaster and D. simulans species despite rapid sequence-level turnover of centromere DNA and the discovery of truly telocentric chromosomes in D. sechellia.

Overall, the manuscript is well-written, clear, and the combined use of both cytogenetic and genomic investigations is a strength that renders major findings robust to methodological artifacts or biases. Despite these strengths, I have several major criticisms.

Thank you for your positive comments about the manuscript's clarity and robustness. We address the major criticisms below.

First, the author's primary conclusions are not overtly novel, as they reinforce a trend of rapid centromere sequence turnover that is widespread across eukaryotes. Centromeres of several other taxa are known hotspots for selfish TE accumulation, which further lessens the novelty of the discovery that Drosophila centromeres have been colonized by G2/Jockey-3. While the authors' work helps to establish the precise sequence-level nature of rapid centromere evolution in Drosophila, the recapitulation of broadly established patterns nonetheless diminishes the perceived impact of this work.

While rapid centromere sequence evolution has been described in other species, our work goes beyond previous observations and brings novel insight into centromere evolution for multiple reasons that we list below:

First, most previous studies documented rapid centromere evolution as changes among the same repeat family or over longer evolutionary timescales than the ones we study here (Melters et al 2013). For example, the *alpha*-satellite associated with the centromere in humans is part of a satellite family that originated approximately 65-85 Mya ago (Lee et al 2011, doi: [10.1093/gbe/evr083](https://doi.org/10.1093%2Fgbe%2Fevr083)). This satellite is associated with centromeres across many monkeys and apes, although different sequence variants are present in different species. We make this point in the Discussion lines 488-497:

*“In the last decade, several studies have shed light on the rapid evolution of centromere sequences in a wide range of species [12]. Centromeres are dynamic in their genomic location and can rapidly diverge in sequence between related species. However they generally consist of different variants of the same type of repeat element (either retroelements or satellites) [49–56] therefore maintaining a certain homogeneity among closely related species. For example, human and its closely related species — chimpanzee, orangutan, and macaque — diverged about 7 million years ago but all centromeres are populated by different subfamilies of the 𝛼-satellite repeat [51,52]. Arabidopsis species, A. thaliana and A. lyrata, also experienced a turnover of centromere sequences since their divergence (5-6 Mya), but between related satellites [57].”*

While many comparative studies refer only to DNA sequence changes in centromeric and pericentromeric regions, our study is on centromere organization at both the DNA sequence and chromatin levels. Here, we discover dramatic shifts in centromere composition that are categorical — from retroelements to satellites, back to retroelements — and occur on a very short timescale (in <2.4 My and <240,000 yrs) highlighting extreme centromere evolutionary dynamics. We clarify this point in the Discussion lines 497-510:

*“In this study we reveal that Drosophila centromeres appear to experience recurrent turnover between different repeat types over short evolutionary timescales (Fig7). We hypothesize that the ancestral centromeres resembled the retroelement-rich islands of D. melanogaster and that centromere turnover in the D. simulans clade species was facilitated by the rapid spread of the 500-bp and 365-bp complex satellite repeats (<2.4 Mya). The only retroelement countering the domination of these complex satellites and preventing the complete homogenization of centromeres is G2/Jockey-3. Following the emergence of the centromeric complex satellites, the centromere shifted to the neighboring telomeric HTT in D. sechellia on the X and dot chromosomes (in <240 Kya). This rapid evolution of centromere sequences seems to be a general feature of the Drosophila genus [58]. One clade where centromere evolution seems to experience similar dynamics is in the Equus genus, where evolutionarily new centromeres appear in chromosomal regions free from satellite DNAs (e.g.,[59]).*

Second, we discovered that this shift happened *within* species. In *D. sechellia*, the X and 4 chromosome centromeres have shifted to retroelements specialized in telomere functions, while the other centromeres remain associated with complex satellites. This also reveals that the centromeres and telomeres can occupy the same array of repeats that now need to perform both essential roles. This is discussed at line 512-535.

*“The X and dot chromosomes of the melanogaster species are classified as acrocentric based on cytological observations of mitotic chromosomes (reviewed in [43]). Here, our epigenetic profiling and high-resolution cytology allows us to distinguish between chromosomes with independent, but nearby centromere and telomere domains (e.g., in Mus Musculus where centromeres are positioned 10–1,000 kb away from the telomere [60,61]), and telocentric chromosomes where centromeres and telomeres are on adjacent sequences (e.g. Mus Pahari [45]) or both occupy the same repetitive array. While the centromere shift to the HTT could be a cause or consequence of the loss of the centromeric satellite, the presence of 500-bp satellite adjacent to the telocentromeric domain on the X chromosome (Fig 4A-C) suggests the latter scenario. We therefore suspect that the association of the HTT retroelements and the centromere is due to centromere shift rather than centromere-targeted transposition. While in D. sechellia X and dot chromosomes are clearly telocentric, we think that centromeres are close to the telomeres in D. simulans and D. mauritiana. Our observations raise important questions regarding the respective roles of centromeres and telomeres in chromosome biology as well as their functional association. Interestingly, in fission yeast the telomere bouquet is essential for spindle formation through telomere-centrosome contacts. However, if the telomere bouquet is disrupted, centromere-centrosome contacts can rescue the spindle defect, suggesting that centromeres and telomeres have functional similarities and interchangeable roles [62]. Similarly in mice, one of the shelterin complex proteins that is essential for telomere function (TRF1) is also required for centromere and kinetochore assembly [63]. In the case of D. sechellia, HTT elements with historical telomere-specific functions now need to also carry out and avoid interfering with centromere functions, at least at the structural level.”*

Third, our comparative genomic, phylogenetic and cytogenetic analyses suggest that a centromere-associated retroelement, *G2/Jockey-3*, is abundant in these species and selfishly targets centromeric chromatin rather than a target DNA sequence across species. Interestingly the colonization pattern of *G2/Jockey3* appears different in the 4 species. Thus the *melanogaster* clade is an excellent model to study the dynamics of centromeric-associated retroelement. See Discussion lines 537-559.

*“Although the dot and X centromeres of D. sechellia are unique due to their association with telomere-specialized retroelements, transposable elements (TEs) are commonly found in the centromeres of the simulans clade, even when satellite DNA is the predominant repeat. G2/Jockey-3 seems to have actively targeted centromeric regions in the ancestor of D. melanogaster and the D. simulans clade, despite their disparate underlying sequence composition. This suggests that this element may target centromeric chromatin itself rather than a specific sequence. Such centromere-chromatin targeting by retroelements may also exist in maize [64,65] and Arabidopsis [57,66,67]. Transformation experiments in Arabidopsis showed that the centromere-associated Tal1 retroelement from A. lyrata is able to target A. thaliana centromeres [66] despite divergent (30%) centromeric satellites in these species [68].*

*On one hand, TEs may limit harm to their host by inserting at centromeres, far from protein-coding genes and with little opportunity for deleterious ectopic recombination [28,69,70]. They may also escape host defenses by inserting in CENP-A nucleosomes [71]. However, a high density of TEs may inactivate centromeres through heterochromatinization [27,72]. On the other hand, centromeres may tolerate TEs that contribute positively to a proper chromatin and transcription environment for centromere assembly, and in a sense therefore cooperate with the genome. Indeed, there is evidence across species that RNA is important for centromere assembly [73–77]. Centromeric copies of G2/Jockey-3 are transcribed in D. melanogaster [29], therefore these TEs might contribute to centromere function despite having properties of an opportunistic selfish genetic element.”*

Finally, we propose that the rapid centromere evolution in these species is due to genetic conflicts over transmission through the female germline. Retroelements and satellite DNA may indirectly compete for centromere occupancy. This is supported by the Y chromosome centromeres: the one genomic location where we do not see categorical shifts in centromere occupancy, and the only chromosome which never experiences female meiosis. We discussed this point lines 598-606:

*“The unique composition of Y chromosome centromeres, where we do not observe centromere turnover, may be because it is the only chromosome that never experiences female meiosis (Fig 7B). While selfish centromere drivers (e.g., driving satellites) cannot invade Y chromosomes, these chromosomes still offer a safe haven for transposable element insertions. However, Y chromosomes are subject to different evolutionary pressures and mutation patterns that might affect its sequence evolution [34], although not exclusively at the centromere. Distinguishing between drive and any alternative hypotheses will require future empirical studies of chromosome transmission and the development of formal population genetic models for centromere drive.”*

We therefore believe that our findings are novel and have broad implications for centromere biology and genome evolution.

A second major set of concerns focus on the approaches used for CUT&Tag data analysis:

(1)     Important control experiments are missing. For one, the authors do not present results that demonstrate specificity of their custom-generated CENP-A antibody across all samples and species. This validation step is crucial for establishing that observed signals of CENP-A association are biological, not artifactual. Second, the authors do not present any data to establish that their CUT&Tag libraries are enriched for ~130bp CENP-A nucleosomes. For example, TapeStation results would demonstrate proper isolation of mononucelosomal DNA across all samples and confirm the expected ~20bp size shift between CENP-A and H3K4me3 (positive control) nucleosomes. These control experiments are vital for establishing the quality of their CENP-A CUT&Tag libraries.

We regret that we did not elaborate on the validation of our custom-generated CENP-A antibody. To validate the specificity of our custom-generated CENP-A antibody compared to the commercial CENP-A antibody (Active Motif) used in a previous study (Chang, Chavan, Palladino et al. 2019), we used two different approaches:

1. We performed Western blots on samples from all 4 species using our custom-generated CENP-A antibody. We observed a single band at the expected size of around 38 kDa. We now include the result of the Western blot in S11 Fig.

We mention this validation in the Material and Methods lines 662-663.

*“We validated the specificity of the antibody by Western blot (S11 Fig)”*

and lines 688-700.

“***Western blot***

*Twenty flies from each species were homogenized in 200µl 1x Laemmli buffer (diluted from BioRad 4x Laemmli Sample Buffer [1610747] with 2-mercaptoethanol [Sigma] and 1x Pierce EDTA-free Protease inhibitors [ThermoFisher A32965]), denatured by incubation at 95C for 10 minutes, centrifuged at 15000 rpm for 5 minutes at 4C, and 20µl of each the supernatant and PageRuler Prestained Protein Ladder (ThermoFisher [26616]) was run 4-15% Mini-Protean TGX gel. The protein was transferred to PVDF membrane (Novex Invitrolon [LC2005]), blocked (Li-Cor Intercept Blocking buffer [927-60001]), incubated with 1:1000 Rabbit anti-CENP-A(lab stock), washed 3 times with TBS/0.1% Tween-20, incubated with 1:20000 Goat Anti-Rabbit IgG (H+L) DyLight800 (Invitrogen SA5-10036), washed 3 times with TBS/0.1% Tween-20, and imaged with Li-Cor Odyssey CLx imaging system.*”

2. We previously identified the *Drosophila melanogaster* centromeres using ChIP-Seq with Actif Motif CENP-A antibody (Chang, Chavan, Palladino et al. 2019). To validate our custom-generated CENP-A antibody we repeated that experiment, by performing CUT&Tag in *Drosophila melanogaster*. We recapitulated the same results we previously had with Active Motif CENP-A antibody. The results are presented in S5 Fig.

*“We validated our approach with CUT&Tag in D. melanogaster, which recovered the same centromere islands as in Chang, Palladino, and Chavan et al. [30] (S5 Fig).“*

In addition, we now include an example of the bioanalyzer profile of the CUT&Tag libraries we obtained for our custom-generated CENP-A and H2K27me3 antibodies in supplemental S11 Fig, see lines 732-734.

*“We analyzed each library on Bioanalyzer for quality control, representative profiles of CENP-A and H3K27me3 profiles are provided in S11B Fig.”*

Unfortunately, because of the relatively low abundance of CENP-A chromatin, a low concentration, and thus a large fragment size distribution for the CENP-A, we do not have the resolution to distinguish a shift between the first peak of CENP-A and H2K27me3.

(2)     The authors sequence their CUT&Tag libraries using pe150 reads. Given that the length of a CENP-A nucleosome is <300bp, this strategy will result in double-sequencing of nucleotides in the middle of each sequenced fragment. It does not appear that this region of overlap from forward and reverse strands was bioinformatically removed during the authors' data processing steps. This could lead to false inference of CENP-A association peaks.

Thank you for pointing out that we need to clarify our experimental design. In this experiment, we are not aiming for nucleosome resolution, which might actually be counterproductive here. Because of the repetitiveness of the region, having fragments of 130 bp would make it difficult to map these reads uniquely to the genome. Our bioanalyzer results (S11B Fig) indicate that the fragment size goes over 300 bp. We thus chose to make 150-bp paired-end libraries to increase our ability to map reads uniquely. We used MACS2 to call the CENP-A-associated peaks. In paired-end mode, MACS2 takes into account the fragment length of paired reads, thus even if the reads partially overlap, they will not be counted twice. We have included an explanation of the fragment sizes in the methods section, line 1230:

*“We called peaks based on fragment size using MACS2 callpeak [85] (v2017-10-26) (option -f BAMPE -g dm -q 0.01 -B --call-summits) and performed an IDR analysis (https://github.com/nboley/idr) to identify high confidence peaks that overlapped between replicates (IDR <0.05, S1 Table).”*

See Gaspar 2018 for improved peak calling in MACS2 paired-end mode: <https://www.biorxiv.org/content/10.1101/496521v1.full.pdf>

(3)     Unlike ChIP-seq, there is no chromatin "input" in CUT&Tag that can be used to normalize data for inter-sample comparisons. The current recommended work-around involves normalization to a spike-in control DNA sample from a divergent source (see Epicypher website). Instead, the authors normalize their data to RPM, a strategy that does not account for differences in chromatin capture biases across samples or differences in antibody specificity. Thus, the method of data normalization does not allow for straight apples-to-apples analyses across samples/species, as the authors perform. For example, differential CENP-A association with specific sequences/repeat elements could simply reflect systematic differences in pA-Tn5 chromatin accessibility across samples, rather than genuine species-level differences in centromere sequence identity. While cytogenetic experiments bolster conclusions from CENP-A CUT&Tag analyses, some discussion of the limitations of the CUT&Tag methodology is warranted.

We agree that the spike-in control would be useful to measure quantitative differences between samples, however this is not our objective. We decided not to include a spike-in in our experiment for technical reasons. Unlike other histone marks, CENP-A has a very low abundance, resulting in an extremely low DNA output after CUT&Tag, making the sequencing challenging. We worried that including a spike-in would result in an overrepresentation of our spike-in DNA versus CENP-A associated DNA. To maximize our read recovery, we thus decided not to include a spike-in.

If we were making quantitative claims about the variation in CENP-A between species, the spike-in would be helpful to normalize the efficiency of the assay across samples. Here we do not make such quantitative claims, instead, we focus on qualitative variation: *e.g.,* which repeats CENP-A binds to in different species, which we then validate through cytogenetics. In Figure 1, we show the most enriched sequences within each sample. We agree with the reviewer that CUT&Tag does have its limitations, and we are restricted in terms of the resolution that we can obtain, but this does not change our conclusions. We clarify this point in lines 726-728:

*“While a spike in control would allow us to measure quantitative variation between samples, our analysis of centromere chromatin is qualitative. We therefore elected to exclude a spike in to maximize our centromere-associated read recovery.”*

(4)     Both main and supplemental figures zoom in on centromere regions, excluding other genomic windows. This makes it difficult to appreciate how CENP-A enrichment signals differ between centromeric and non-centromeric regions. Is it the case that signal is restricted to centromeric contigs, to the exclusion of other genomic positions?

We agree we should have been more explicit about where CENP-A is enriched across the genome. The CENP-A enrichment signal is indeed restricted to the centromere regions that we highlighted in the figures. In Supplemental Figure 4, we plotted the result of the IDR analyses, showing the localization of the peaks that are consistent across replicates. The enriched CENP-A regions are almost exclusively localized on the contigs corresponding to the centromere candidates. There are a few loci enriched for CENP-A outside of the centromeric contigs, but these are usually small and correspond to repeats that are also present at the centromere, such as *G2/Jockey3* (S1 Table). We clarified this point in lines 164-168:

*“For each species, there were five contigs that were consistently among the most CENP-A-enriched contigs (S4 Fig), which we considered to be the centromere candidates for each chromosome (S2 Table). We found almost no consistent CENP-A signal outside of these centromere candidates (S4 Fig, S1 Table).”*

Third, the observation that CENP-A localizes to HTT sequence leads the authors to conclude that the X and dot chromosomes are truly telocentric in D. sechellia. However, it appears that distinct sub-domains within the HTT sequence array are potentially serving telomere and centromere-like functions. For example, the first peak inferred from uniquely mapped reads on chr4 is at 40kb. To me, it seems just as likely that there are subtle differences in chromatin environment that compartmentalize the HTT array into sections that execute telomere- and centromere-functions, rather than a single domain that performs both functions simultaneously, the interpretation the authors seem to favor. In the absence of functional experiments demonstrating co-localization of telomere/centromere functions to the same locus, I'm not convinced that the authors can firmly distinguish between the nearly vs. truly telocentric hypotheses.

We agree with this comment. Despite knowing that both centromere and telomere share the same type of sequences, we do not have the ability to determine if there are two separate domains or if they overlap. We are now working on the experiments which would allow us to distinguish between these hypotheses but this is beyond the scope of this paper. We clarify that point in lines 361-364:

*“We demonstrate here that centromeres can share sequence components with telomeres [45]. Currently, we lack the ability to ascertain whether the centromere and telomere share a common domain or exist as separate domains within the HTT array.”*

I also have some more minor critiques :

*      CENP-A peaks from single and multi-mapping methods are quite distinct, with many peaks observed in only one of the two methods of data analyses. There is no discussion of these differences, or whether one set of peaks may more precisely or more comprehensively define the active centromere. Overall, I think the paper would benefit from some guidance to the reader on how to interpret the differences in the two sets of peaks.

Thank you for making this point. The two types of peaks are actually overlapping. That is, the majority of the peaks from the uniquely mapping analysis (in black) overlap with the peaks from all reads (including multi-mappers) analysis (in gray), and so when they overlap, only the reads/peaks from the uniquely mapped analysis are visible. So any differences in enrichment shown in gray are specific to the all-read (including multi-mappers) analysis. The precise locations of the peaks from each analysis are indicated in supplemental table S1.

We clarified all of these points in the legends of Figures 1-5 and S1-S3, S5.

*“Black and gray plotted lines represent the enrichment based on uniquely mapping and all reads (including multi-mappers), respectively. The black and gray tracks below each plot correspond to MACS2 peaks showing significantly enriched regions based on the uniquely mapping and all reads (including multi-mappers), respectively. The precise locations of all peaks are listed in Table S1.”*

*       What is the rationale for performing IF-FISH studies with CENP-C, as opposed to CENP-A?

We choose to use CENP-C instead of CENP-A for IF-FISH on mitotic spreads due to a technical limitation. To obtain high-quality chromosome spreads, we use acetic acid, which also removes histones. Thus, it is not feasible to perform IF on mitotic spread using anti-histone antibodies, such as anti-CENP-A. As CENP-C overlaps with CENP-A in *D. melanogaster* (Przewloka et al 2007, <https://doi.org/10.1371/journal.pone.0000478>), we elected to use CENP-C as a centromere marker for validation on mitotic chromosomes. We added this point in the methods, lines 843-846:

*“We use acetic acid to obtain high quality chromosome spreads, however this also removes histones. Thus, it is not feasible to perform IF on mitotic spread using anti-histone antibodies, such as CENP-A. We therefore use CENP-C,–a kinetochore protein that marks centromeres and overlaps with CENP-A [38].“*

and the result line 194-197:

*“We confirmed the localization of centromeric protein CENP-C, a kinetochore protein that marks the centromeres and has documented overlap with CENP-A [38], on the 500-bp (Fig 1C, F, and I) and 136-bp (S6A Fig) satellites.“*

*       Is there homology between 365-satellite and 500 satellite? Is it possible that 365-satellite derived from a deletion and subsequent divergence from 500-satellite?

Thanks for raising these questions, as it was not clear in the text. There is no homology between the 365bp and the 500bp satellite, they appear to have independent origins. We now make this point clear in lines 260-261:

*“The 365-bp satellite shares no homology with the 500-bp satellite, suggesting an independent origin.”*

*       Typos on lines 533 and 537: "synthesis" -> "synthesized"
*       Typo on line 577: "Epycpher" -> "Epicypher"
*       There is inconsistent use of CENP-A versus Cid throughout the text (e.g., lines 578, 619).
*       Similarly, there is inconsistent use of G2/Jockey3 versus G2/Jockey-3 in the text and figures.
*       136-satellite is referenced as 135-satellite in the figure legends. This difference should be reconciled for consistency.
*       Sup Tables 1, 3, 4, and 5 need column names. The data are not otherwise interpretable.

Thanks for making these points, we made all of the suggested corrections.


Reviewer #2:

The manuscript by Larracuente and colleagues addresses the evolution of centromeres in four Drosophila species. The four species include three closely related species from the 'simulans' clade and the more distantly related classic model system D. melanogaster. A reason the evolution of centromeres is of particular interest is its epigenetic nature. During development and through sexual reproduction, centromeres are reliably inherited. However, over longer evolutionary timespans (and in several pathological conditions, such as cancer) the DNA sequence that functions as a centromere can change. In turn, the sequences that underly the genomic region of the centromere are rapidly evolving. However, we have a limited understanding of the specific steps mediating these large-scale evolutionary changes and the forces that drive them.

My main concern about this paper is the poor delineation of the changes that occur to the cis-sequences (the repeated sequence in the region where the centromere is) and the shifts in the region that functions as a centromere (defined as sequences that associate with centromeric proteins CENP-A/CID and CENP-C). This confusion infiltrates all aspects of the papers. For example, it is not clear whether the genome assemblies used by the authors are a contribution of this work or of previous work by the authors that focused on the evolution of the Y chromosomes (eLife 2022). Related to that: what is the quality of this genome assembly and how far is it from proper T2T assembly?

The regions that are shown in the figures - how big are they in relation to the heterochromatic middle of the chromosomes?

How much of the changes reflect a shift of the CENP-A-positive region, and how much is a change in the total sequence in the heterochromatic core of the chromosome? What are the underlying assumptions about the reciprocal relationship between genetic and epigenetic alterations?

We hope that our revisions to the text now make our position on the relationship between changes at the DNA sequence and at the chromatin levels clearer. We address each comment below:

The genome assemblies used in this paper are from previous work (Chang et al., eLife 2022), and we clarify this point on line 151-153.

*“The resulting reads were mapped to versions of each species’ genome assembly with improved representation of heterochromatic regions from previous work [34].”*

These genome assemblies are not T2T (they are not yet available in Drosophila) but they are the most complete assemblies so far. Compared to other assemblies, they have an increased representation of heterochromatin regions, which makes them the most appropriate to work with for this paper.

In Drosophila the pericentromeric and centromeric heterochromatin totals approximately 60Mb (Hoskin et 2002, https://doi.org/10.1186/gb-2002-3-12-research0085). In the *simulans* clade, each centromeric domain spans between 50kb and 200kb, a very small fraction of the heterochromatin region of the chromosome. We now mention this information in lines 182-186:

*“In D. melanogaster, the heterochromatin domain makes up approximately 60Mb of the genome [35], of which centromeres only represent a small fraction (1/200th [30]). In the simulans clade centromeres, the CENP-A domain appears restricted to a 50-kb to 200-kb subset of the 500-bp satellite array (Figs 1A, D, G and 2).”*

The variation in sequence composition extends beyond the centromeric region, encompassing both centromeric and pericentromeric regions across species (Charkraborty et al., 2021). This indicates a dynamic turnover of DNA sequences in and around the centromeres. However, discerning whether these changes occur within a stable CENP-A domain or if CENP-A relocates to new sites remains challenging. Likewise, it's unclear whether alterations in DNA sequences prompt shifts in CENP-A localization (e.g., structural modifications) or if CENP-A relocation leads to the loss of formerly centromeric DNA sequences. Our study can only conclude that the DNA sequences associated with CENP-A chromatin turn over rapidly. We now clarified this point in the Discussion on lines 591-596:

*“The spread of 500-bp to what is now pericentromeres may be a signature of past expansion – CENP-A may have restricted its domain to a subset of the 500-bp satellite array to avoid centromere asymmetry. However, whether these changes occur within a stable CENP-A chromatin domain that the 500-bp and 365-bp complex satellites invaded, or CENP-A relocated to new sites that contained 500-bp and 365-bp complex satellites remains an open question.”*

At the level of language: the terms used in the manuscript are confusing and conflate these two effects. Particularly, the term 'turnover' in the title and throughout is vague and should be replaced with a term that clearly describes what is being found. (Similar problem related to other verbs that suggest dynamics, like 'invaded'.)

Thank you for pointing out that our language was confusing. We agree that it is important to be clear about what we mean by ‘turnover’. We now use words like “emergence” to describe our observation that satellite DNAs specific to the *simulans* clade species now occupy their centromeres. However, we elected to keep the term ‘turnover’, as this term describes the sequence evolution without implying a particular mechanism or cause.

As noted above, the general question of centromere evolution is interesting and, with the recent availability of long-read sequencing, timely. However, the lack of clarity in writing and presentation makes it very challenging to judge the potential impact of a revised manuscript. In other words, it is challenging to tell what will be the impact of a heavily-edited manuscript.

We hope that our revisions focusing on improving clarity throughout the manuscript addresses these concerns.

Other major issues:

The authors invoke a competition between satellite sequences and TEs. Yet it is not clear why satellite DNA would be in conflict with TEs when both could ostensibly exist on the same chromosome and contribute to CENP-A recruitment. A further explanation of this idea is warranted as it is the basis for the model in Figure 7b.

We agree with this comment, as we do not think that there is necessarily a direct competition between retroelements and satellites, as they indeed coexist at centromeres and may contribute to centromere function. We suggest that it is the tendency for selfish behavior of both satellites and TEs (independently, through different modes) that drives centromere turnover. We clarified this point in lines 566-571:

*“While there might not be direct competition between retroelements and satellites, both can coexist and cooperate to allow or even facilitate centromere function, centromeres may then cycle between retroelement-rich and satellite-rich domains through repeated bouts of retroelement invasion, followed by satellite birth and satellite expansion events (Fig 7B). The centromeres that we study here might represent different stages of this cycle.”*

Including the species names in the model in Figure 7b does not make evolutionary sense since all four are extant species and their centromeres do not morph between species as is implied by Fig. 7b. This figure should be changed to include appropriate ancestral states or have the species designations removed entirely.

By including the names of each species in our model of centromere turnover, our intention was solely to illustrate that they represent different stages of cycle, rather than imply any directionality. To clarify this point, we updated the figure and the legend (lines 627-636):

*“An evolutionary model for the centromere sequence turnover in the melanogaster clade species representing the cycling between retroelement-rich and satellite-rich centromeres in the D. melanogaster clade species. Retroelements and satellites may be engaged in their own conflicts and thus indirectly compete to occupy centromeres. Representative examples of specific replacement events in different stages of the conflicts are depicted in the outside circles. For example, while D. melanogaster centromeres are rich in transposable elements, D. simulans clade centromeres are now primarily occupied by satellite DNA. The satellite-rich centromeres of D. simulans are still targeted by G2/Jockey-3 retroelements and D. sechellia’s X and dot (4th) chromosome centromeres shifted to the specialized telomeric HTT retroelements. “*

The categorization of centromeres, other than those of budding yeast, as genetic or epigenetic is an oversimplification that confuses the framework of this manuscript. Although centromeres can form on non-satellite DNA, many studies suggest a genetic contribution to centromeres in eukaryotes. In particular work on the role of CENP-B in human centromeres (https://urldefense.com/v3/__https://doi.org/10.1016/j.devcel.2015.03.020__;!!CGUSO5OYRnA7CQ!fs4GCXBoH4T3f0nMH3qugldZrtED_jZQFOePMHenGRTgL7_9SnwjD1HiFstJj9JMBo-_ukTt59bIQIYtVo7aU3B9$ ) and studies in mice where more satellite DNA recruits more CENP-A  (https://urldefense.com/v3/__https://doi.org/10.1016/j.cub.2017.06.069__;!!CGUSO5OYRnA7CQ!fs4GCXBoH4T3f0nMH3qugldZrtED_jZQFOePMHenGRTgL7_9SnwjD1HiFstJj9JMBo-_ukTt59bIQIYtViaEGsuv$ ) challenge the idea that centromeres are completely epigenetic. Even in Drosophila, there are sequence features that tend to occupy centromeric DNA (e.g. AA dinucleotides). Therefore, it is confusing for the authors to say "In eukaryotes, centromere identity is defined epigenetically" (line 52). This confusion is compounded when the authors later invoke centromere drive as an explanation for the rapid evolution of centromeric DNA sequence. In the centromere drive hypothesis, centromeric DNA acts selfishly to bias its transmission into the egg in female meiosis I. The model assumes that something inherent to the centromeric DNA determines centromere strength. It is essential to delineate the paradigm used in this work - whether it is the centromere drive or a different one.

We regret that we were unclear on this point, as we did not intend to suggest that centromeric sequences play no role in centromere function. On the contrary, the evidence that these sequences indeed can influence centromere function is the motivation for our study, as we explained in both the introduction and in the discussion. However, the specific role of centromeric DNA is not well understood, as DNA sequences evolve rapidly and centromeres can form *de novo* (as in human neocentromeres), leading to the well-established model that centromeres can be more reliably identified by their epigenetic characteristics rather than their sequence composition. Stating that centromeres are defined by CENP-A does not imply that the underlying DNA sequence does not play a role. We changed our wording to make this clearer in the introduction on lines 60-67:

*“In eukaryotes, centromeres are generally marked epigenetically by the presence of the centromere-specific histone H3 variant CENP-A (also known as CID in Drosophila) [2–4]. CENP-A plays a central role in centromere identity and function, where it recruits kinetochore proteins, forming a macromolecular structure that allows spindle microtubule attachment [3]. The overexpression of CENP-A is associated with various cancers in humans [5]. The role of the underlying DNA in centromere function is not well understood, although some sequence properties or abundance may contribute to centromere specification and strength (e.g., [6–8]).”*

The cytological experiments are aimed to "validate" the CUT&TAG experiments, but it is not clear what is being tested, and what means are used to quantify the results and test for significance. The claims based on the mitotic chromosomes should be spelled out and addressed with appropriate quantifications and statistical tests (e.g., to what extent do the centromeric sequences co-localize with CENP-C? Are there any sequences in the heterochromatic middle of the chromosome that DO NOT co-localize with CENP-C?). As it stands, the only thing the IFs on the mitotic chromosomes are validating is the genome assembly - i.e., the presence of the repeated units in the general vicinity of the central constriction of mitotic

chromosomes.

We used recent genome assemblies for three species and identified contigs highly enriched in CENP-A. To validate the sequences contained in these contigs are indeed centromere-associated, we design specific FISH probes and we show signal overlap with the active centromere marker, CENP-C. We more explicitly explain the rationale for these validations in lines 192-194.

*“To validate that the 500-bp and 136-bp satellites are associated with the centromere, we used a cytogenetic approach with IF-FISH on mitotic chromosome spreads from larval brains using Oligopaints targeting each complex satellite [37].”*

Mitotic spreads afford limited resolution, so indeed these experiments simply confirm that our candidate centromere contigs are in the vicinity of the centromere. Therefore, quantification based on these images would not be meaningful. Additionally, as demonstrated by CUT&Tag and the genome assembly data, the *500-bp* satellite is not specific to the centromere; it is also present in the proximal pericentromere. Consequently, as anticipated, we observe signals for the *500-bp* sequence outside of the CENP-C domain. However, the CENP-C signal overlaps the *500-bp* satellite in 100% of the spreads. We clarify this point in the text in lines 197-201.

*“Because mitotic spreads offer limited resolution, it is challenging to distinguish between the centromeric and proximal pericentromeric domains. However, the 500-bp signal extends beyond the CENP-C domain, indicating its presence in both the centromeric and proximal pericentromeric regions, consistent with our genome assemblies and CUT&Tag data.”*

An exception to the previous point is the chromatin fiber experiments (Figure 4D), where the data is clear. However, even these data should be quantified. Also, what is the DNA sequence distal to the HTT signal? It should be commented on given that a major conclusion of the paper is the "truly telocentric chromosomes observed for the first time in these species" (lines 285-286).

To quantitatively validate the association between CENP-A and centromeric sequences we quantified the percentage of CENP-A signal that overlaps with either the *HTT* or the *500-bp* signal. For each chromosome we quantified between 15 to 20 fibers, the measures are now provided in S6 Table. As expected for the *D. sechellia* X chromosome, 89.8 ± 19.4 % of the CENP-A signal overlaps with the *HTT* signal, while only 6.2 ± 13.6 % overlaps with the 500-bp signal. We present these results on lines 331-349.

“*To visualize these regions at higher resolution, we performed IF-FISH on stretched chromatin fibers using a CENP-A antibody and Oligopaints targeting the 500-bp satellite and the HTT elements. These fibers confirm that CENP-A nucleosomes are seated on the HTT domain, and are flanked by, but do not overlap, the 500-bp satellite (Fig 4D). On average, 89.82 ± 19.4% of the CENP-A signal overlaps with the HTT signal, while only 6.2 ± 13.6% overlaps with the 500-bp signal (S6 Table).”*

*…*

*We also observed patterns from stretched chromatin fibers consistent with our predictions for the other chromosome centromeres (S8 Fig). On the dot chromosome 73.02 ± 32.76% of the CENP-A signal overlaps with the HTT signal, with no 500-bp signal nearby (S8 Fig, S6 Table). On the autosomes, 100 % of the CENP-A signal overlaps with the 500-bp signal (S8 Fig, S6 Table).”*

Regarding the lack of FISH signal distal to the *HTT* in Figure 4, we cannot determine if this is a technical artifact or if a small number of different repeats exist more distal to *HTT*. We occasionally observe discontinuity of the FISH signal on fibers, which we think is due to loss of probe binding or failure to bind; this complicates a more precise interpretation of the FISH signal. Since the *HTT* signal extends to the end of the fibers in different images (see example in Supplementary Figure 8), we believe it is more likely due to this technical artifact. We mention this possibility in the text lines 338-343:

*“In some fibers, we observed a lack of CENP-A/HTT signal at the very ends, similar to what we show in Fig 4C. It is possible that there is a small amount of non-HTT sequence distal to the HTT signal on these chromatin fibers. However, we believe that the absence of HTT signal at the fiber ends is likely a technical artifact due to the loss of the FISH signal, as this observation was variable across fibers (see S8 Fig).*

The claim on lines 113-114, regarding male versus female evolution, is premature and unsupported since it is based on an N=1 (i.e., the Y chromosome). While an interesting, speculative discussion point, it is not more than that.

We agree that our observations regarding the Y chromosome, the only 1 (of 5) chromosome that is not transmitted through the female germline, offers indirect support of the model. We softened our interpretation in the discussion and now include alternative hypotheses. We feel that this is an appropriate speculation that is consistent with our observations. See changes on lines 598-606:

*“The unique composition of Y chromosome centromeres, where we do not observe centromere turnover, may be because it is the only chromosome that never experiences female meiosis (Fig 7B). While selfish centromere drivers (e.g., driving satellites) cannot invade Y chromosomes, these chromosomes still offer a safe haven for transposable element insertions. However, Y chromosomes are subject to different evolutionary pressures and mutation patterns that might affect its sequence evolution [34], although not exclusively at the centromere. Distinguishing between drive and any alternative hypotheses will require future empirical studies of chromosome transmission and the development of formal population genetic models for centromere drive.”*

Reviewer #3:

This paper reports a thorough characterization of centromere DNA sequences in drosophila species. As with other centromere genomics studies, it shows rapid changes in centromere DNA. In this case the changes include transitions between retroelements and satellites. The data are interpreted as consistent with female meiotic drive as the evolutionary force responsible for rapid changes in centromere DNA. This interpretation is indirectly supported by the finding that the Y chromosome does not show such high turnover of centromere sequences. Overall, the paper is well written, and the data are convincing. To strengthen the manuscript, the authors might comment on how they envision that centromere DNA sequences could act selfishly in female meiosis. There is no evidence for any functional role of specific DNA sequences at drosophila centromeres (and the authors argue that centromere retroelements also do not have any functional role). How could any DNA sequence gain a functional
advantage in female meiosis, and how might such ideas be experimentally tested in future studies?

Thank you for your positive comments. We added a paragraph in the discussion regarding the potential mechanism through which centromere sequences could drive during female meiosis on lines 586-591.

*“However, it is also possible that the repeats spread through selfish means: for example, if expansions of these repeats corresponded to stronger centromeres that became selfish and could drive in female meiosis. Repeat expansions may be accompanied by the accumulation of centromeric chromatin, thus recruiting more kinetochore proteins and biasing their segregation to the oocyte, as is the case for the minor satellite at mouse centromeres.”*

Reviewer #4:

Courret and colleagues have investigated the structure of centromeres in several Drosophila species that are closely related to Drosophila melanogaster.  Members of this team have previously determined the centromere structure of D. melanogaster, finding that retrotransposons rather than satellite DNAs form the centromeres.  That was a landmark study and this paper shows a similar high quality.  There are a few major points to address.

1) Evidence for telocentric chromosomes.

a) Have the authors validated that the HTT arrays are truly at the end of the chromosomes 4 and X in sechellia?  This is important since telomere structures can also turn over rapidly in Drosophila (10.1101/gr.245001.118).  Fig 4D seems to show DAPI signal distal to the HTT signal.

The presence of the *HTT* at D. *sechellia* telomeres was assessed by Berlocco et al 2015 using polytene chromosomes. We confirm that the *HTT* are indeed at the end of chromosomes 4 and X on our mitotic spreads (see Figure 4) and our fiber FISH shows that fibers end at or near the *HTT* signal. We think that the lack of *HTT* signal at the end of the fiber is a technical artifact as not all the fibers show this pattern (see supFig8 and response to reviewer 2); FISH signals are usually not continuous on fibers. We cannot exclude the possibility that there is another unknown sequence after the *HTT* domain, although it would likely only be a small region based on our and others’ cytology. Only a true T2T assembly would allow us to confirm this observation at the nucleotide level. Either way, the observation that CENP-A sits on distal *HTT* elements that appear to be at the telomere ends of the chromosome is an important and interesting observation.

We mention this possibility in the text lines 338-343:

*“In some fibers, we observed a lack of CENP-A/HTT signal at the very ends, similar to what we show in Fig 4C. It is possible that there is a small amount of non-HTT sequence distal to the HTT signal on these chromatin fibers. However, we believe that the absence of HTT signal at the fiber ends is likely a technical artifact due to the loss of the FISH signal, as this observation was variable across fibers (see S8 Fig).”*

b) All chromosomes have two telomeres.  Has the centromere moved to the nearby chromosome end, a "shift", or has it moved to the more distant end, a "jump"? 

Thank you for this question. Given the presence of the *500-bp* satellite next to the CENP-C domain, we think it’s more likely a ‘shift’ to the nearby telomere. We now make that point in the Discussion lines 518-521.

*“While the centromere shift to the HTT could be a cause or consequence of the loss of the centromeric satellite, the presence of 500-bp satellite adjacent to the telocentromeric domain on the X chromosome (Fig 4A-C) suggests the latter scenario.”*

c)  Related to above point, it would be useful for the authors to diagram the chromosome structures. For example, Fig 4A shows that the HTT array is adjacent to the 500 bp array in sechellia. Is that also true in simulans and mauritiana, or was there a re-arrangement or internal deletion that brought those two arrays close together in sechellia?  Have there been any large-scale rearrangements of X and IV between the species?

Thank you for the excellent suggestion. We have now included an additional Figure (Fig 7a) that provides a schematic representation of each chromosome's structure, highlighting the centromere and pericentromere composition. We hope this clarifies the differences in chromosome structure between species. According to our assemblies, the *HTT* and *500-bp* domains are also close to each other in *D. simulans* and *D. mauritiana*. However, we cannot rule out the possibility of rearrangements between these sequences that may have facilitated the shift of the centromere toward the telomere.

*"Fig 7a: Schematic illustration of the centromere structure and composition in the* melanogaster *clade. Each chromosome's structure is depicted in grey above each column. Below, we provide a detailed view of the centromeric and pericentromeric regions for each species. The centromere is represented as a circle. Each region is color-coded based on the dominant repeat composition, with the legend at the bottom of the figure explaining the color scheme."*

d) Is it possible to generate high-resolution anaphase images that might shed light on the above questions?

This is an interesting idea, but unfortunately, even in anaphase we lack the resolution to distinguish the two domains.

2) Mitotic figures.  The immuno-FISH images are good quality but their resolution is limited and not always accurately described.

L254/255.  The images appear opposite to the description, the 500-bp signal is flanked by the HTT signal.
L344/345.  The G2 signal on X looks partially overlapping the centromere, similar to some other chromosomes in Fig 6, not 'flanking'.

We agree with the reviewer that the resolution of the Immuno-FISH on mitotic spreads limits our ability to distinguish between centromeric and proximal pericentromeric domains. We now clearly state these limitations, see lines 197-201:

*“Because mitotic spreads offer limited resolution, it is challenging to distinguish between the centromeric and proximal pericentromeric domains. However, the 500-bp signal extends beyond the CENP-C domain, indicating its presence in both the centromeric and proximal pericentromeric regions, consistent with our genome assemblies and CUT&Tag data.*

In addition, on line 316-317 we now write:

*“On one HTT-CENP-A enriched contig, the HTT domain is adjacent to the 500-bp satellite, suggesting that it corresponds to the X chromosome centromere (Fig 4C).”*

and on lines 428-429, now we refer to Fig2A instead of Fig 6C.

*“We identified* G2/Jockey-3 *insertions in each centromere except for the X chromosome, where it directly flanks the centromere (Fig 2A).”*

3) Evidence for genetic conflict.  The authors heavily emphasize genetic conflict as the explanation for their results, largely based on the observed patterns of rapid evolution. Same as with protein/DNA evolution, neutrality can also explain rapid evolution and should be the null hypothesis. Satellite abundance can change rapidly due to unequal crossing over.  TE activity may more commonly be selfish, but as the authors note a TE strategy to insert in or near centromeres could reflect TE evolution to minimize negative host effects.

The authors thus need to explicitly state what patterns would distinguish neutral vs selfish hypotheses.  The only argument presented is that the different patterns observed on Y vs other chromosomes could reflect its inability to experience female meiotic drive.  This is an interesting idea though the Y is also unique in only being present as a haploid chromosome, only impacting male fitness and being non-recombining.

Thank you for raising this point. Presently, we do not have a good way of distinguishing between these hypotheses directly using models of sequence evolution. Any experiments (see below) are challenging and as they may be dependent on genetic background. We are currently pursuing both of these angles, but experimentally testing this hypothesis is beyond the scope of this manuscript. We now discuss the neutral hypothesis on lines 583-591:

*“* *It is possible that the rapid turnover of centromeric sequences was neutral, as satellite DNAs are known to rapidly expand and contract through recombination-mediated processes (reviewed in [16]). However, it is also possible that the repeats spread through selfish means: for example, if expansions of these repeats corresponded to stronger centromeres that became selfish and could drive in female meiosis. Repeat expansions may be accompanied by the accumulation of centromeric chromatin, thus recruiting more kinetochore proteins and biasing their segregation to the oocyte, as is the case for the minor satellite at mouse centromeres [8].”*

The authors strongly favor female meiotic drive as being the cause of rapid centromere turnover.  This could be readily tested by examining segregation of these divergent chromosomes in hybrid backgrounds. One such test of divergent chromosome 4s found no evidence for meiotic drive (10.1534/genetics.114.172072).

We agree that assessing chromosome segregation in hybrids presents a direct approach to testing the centromere drive hypothesis, although. In Gilliland et al. 2015, they focused solely on the segregation of the 4th chromosome in sim-mel hybrids. However, to comprehensively evaluate this hypothesis, it is imperative to examine all hybrid combinations and measure the segregation of each chromosome. Since the centromere drive hypothesis suggests that centromere proteins may have evolved to suppress drive, it is possible that the drive could be cryptic in distant species, and the origin of the centromeric proteins could be significant. For instance, placing the *melanogaster* centromere in a *simulans* background might yield different results. Fully testing this hypothesis requires substantial effort, and it is part of our ongoing research plans.

4) Presentation of satellites and TEs.  The authors first focus on the presence of satellites at centromeres on pp. 6-9, citing figures 1-3, and don't mention the G2 elements until p. 13.  However, a lot of the Cenp-A signal in figures 1-3 obviously overlaps with various TEs. I found this confusing when I first read the Results until I got to p. 13.  I'd suggest first giving a brief overview including that much signal was found to map to TEs, and then can come back to G2 evolution later.
For example, L146 says 'mainly comprised of 500-bp satellite repeat' but Figs 1 and 2 show a lot of signal overlap with TEs.

Thank you for the suggestion. We now highlight the presence of TEs at the centromere at the beginning of the results, see line 177-179:

*“While the 500-bp satellite is the primary repeat type in these simulans clade centromeres, they also contain transposable element insertions, including G2/Jockey-3 (Fig 1A, D, G, S1-3 Figs and Fig 2, S1-3 Figs).”*

Minor points.

L102/103.  Are these precisely known dates or estimates?

We now indicate that these dates are estimates.

L105.  "None … are conserved".  The phrasing here implies that the centromeres are not orthologous / have turned over completely?  Is this what the authors mean, or instead that they have more divergence than expected?
Yes we mean that the centromere sequences have turned over completely between *D.* *melanogaster* and the *simulans* clade species.

We clarified this point in lines 223-226 :

*“We discover that there has been a complete turnover of centromeric sequences in the ~2.4 Myr since these species diverged from D. melanogaster: none of the D. melanogaster retroelement-rich centromeres are conserved in the D. simulans clade.”*

L532.  detailed, not 'details'.

Thanks for noticing. We corrected this error.

L667.  Were larvae used all females?

We used both male and female larvae and now indicate this in the text on lines 829-830.

*“We dissected brains from third instar larvae (both sexes) in PBS…”*

Fig 6A.  What is Jockey-1_Dse?

Jockey-1_Dse is used as the outgroup for the phylogeny. We added a small clarification, see lines 802-804.

*“ We did the same with Jockey-1 (LINEJ1_DM) as confirmation of our methods and to use as an outgroup for the TE fragment alignment.”*
